# Unlocking the potential of other effective area-based conservation measures (OECMs) for achieving conservation targets: A global scoping review

**DOI:** 10.1007/s13280-025-02341-3

**Published:** 2026-02-07

**Authors:** Dimitra Petza, Eva Amorim, Emna Ben Lamine, Francesco Colloca, Esther Dominguez Crisóstomo, Erika Fabbrizzi, Simonetta Fraschetti, Ibon Galparsoro, Sylvaine Giakoumi, Maren Kruse, Vanessa Stelzenmüller, Stelios Katsanevakis

**Affiliations:** 1 Department of Marine Sciences, University of the Aegean, University Hill 81100, Mytilene, Lesvos Island Greece; 2 International Estuarine &, Coastal Specialists (IECS) Ltd, Leven, UK; 3 Centre National de la Recherche Scientifique (CNRS), Université Côte d’Azur, 28 Avenue Valrose, Bâtiment Recherche Sciences Naturelles, 06000 Nice, France; 4 Integrative Marine Ecology Department, Stazione Zoologica Anton Dohrn, Via G. Allegri 1, 00198 Rome, Italy; 5 Department of Biology, University of Algarve, Faro, Portugal; 6 Department of Biology, University of Naples Federico II, Naples, Italy; 7 Marine Research Division, AZTI, Herrera kaia portualdea, z/g. 20110, Pasaia, Spain; 8 Department of Integrative Marine Ecology, Stazione Zoologica Anton Dohrn, Sicily Marine Centre, Lungomare Cristoforo Colombo (Complesso Roosevelt), 90149 Palermo, Italy; 9 Thünen Institute of Sea Fisheries, Herwigstr. 31, 27572 Bremerhaven, Germany; 10 National Biodiversity Future Center (NBFC), Palermo, Italy; 11 Department of Marine Ecology, Centro de Estudios Avanzados de Blanes (CEAB-CSIC), 17300 Blanes, Spain

**Keywords:** Area-based management measures, Biodiversity conservation, Convention on biological diversity, Kunming-Montreal global biodiversity framework, Spatial conservation targets, 30 × 30 target

## Abstract

Other Effective Area-based Conservation Measures (OECMs), introduced by the Convention on Biological Diversity (CBD), refer to areas outside formal protected-area networks that deliver effective and enduring in situ biodiversity conservation. This scoping review systematically examined global approaches to identifying and evaluating potential OECMs. Analysing 99 studies covering 694 case studies and 237 000 potential sites, we found that potential OECMs are widespread, particularly in Asia and terrestrial environments, with most initiatives led by the environmental sector. Assessments relied largely on qualitative expert knowledge, with limited application of analytical methods. Although CBD criteria were commonly applied, contributions of related to ecosystem services and socio-cultural values were often overlooked. Effectiveness evaluations showed considerable uncertainty, with over one-third of case studies reporting inadequate evidence of conservation outcomes. The review emphasises the need for standardised assessment methodologies, improved decision-support tools, and socio-cultural integration to enhance OECM recognition, particularly under the 30 × 30 conservation biodiversity target.

**Supplementary Information:**

The online version contains supplementary material available at 10.1007/s13280-025-02341-3.

## Introduction

In response to rapid ecosystem deterioration and escalating biodiversity loss, the global conservation community is considering alternative approaches and introducing new tools to complement traditional, well-established ones, such as protected areas. Other Effective area-based Conservation Measures (OECMs) have gained global recognition as an innovative and potentially transformative development in conservation policy (Diz et al. 2018; Dudley et al. 2018; Jonas et al. 2018; IUCN-WCPA 2019; Alves-Pinto et al. 2021; Gurney et al. 2021; Claudet et al. 2022). Unlike protected areas, which are the cornerstone of conservation efforts (Watson et al. 2016), OECMs achieve conservation mainly as a by-product of other management approaches and goals (IUCN-WCPA 2019).

The term OECM was introduced in 2010 through Target 11 of the Convention on Biological Diversity's (CBD) Strategic Plan for Biodiversity (CBD 2010). The definition, guiding principles, common characteristics, and criteria for identifying OECMs were agreed upon by CBD parties in 2018 (CBD 2018) and reaffirmed at CBD COP16 in 2024 to ensure their effectiveness in delivering tangible benefits for biodiversity. According to the CBD Decision 14/8 (CBD 2018), key criteria for recognising an OECM include a clearly defined geographic area not currently recognised as a protected area (Criterion A), appropriate governance and management (Criterion B), and the capacity to achieve positive and sustained long-term outcomes for biodiversity conservation (Criterion C). These outcomes also encompass associated ecosystem functions and services, as well as locally relevant values such as cultural, spiritual, and socio-economic aspects where applicable (Criterion D). The significance of OECMs in global and regional biodiversity conservation has also been recognised by other major policy initiatives, such as the 2030 Agenda for Sustainable Development (Target 14.5; UN 2015), the Kunming-Montreal Global Biodiversity Framework (Target 3; CBD 2022), and the EU Biodiversity Strategy for 2030 (EU 2021).

Both protected areas and OECMs are expected to contribute to long-term and effective *in-situ* biodiversity conservation (Jonas et al. 2014; Watson et al. 2016; Dudley et al. 2018). However, while protected areas have nature conservation as the primary management objective (Maxwell et al. 2020), OECMs deliver effective in-situ biodiversity conservation regardless of their management objectives and designation rationale (Jonas et al. 2014; Laffoley et al. 2017; IUCN-WCPA 2019). As such, OECMs represent a novel conservation approach where conservation outcomes are incidental to existing spatial management practices. In other words, OECMs are identified and recognised rather than formally designated for biodiversity conservation (IUCN-WCPA 2019; FAO 2022). They encompass a wide range of landscapes and seascapes, including indigenous and community-conserved territories, sustainably managed fisheries-restricted areas, privately owned lands, and sacred sites with cultural significance. Despite their diverse forms and governance structures, all OECMs have in common the potential to deliver effective and enduring biodiversity conservation while supporting ecosystem services, sustainable resource use, and socio-economic well-being (Dudley et al. 2018; IUCN-WCPA 2019; Gurney et al. 2021).

In the years following the OECMs official definition, additional general and sector-specific guidance has been developed by the International Union for the Conservation of Nature (IUCN), the Food and Agriculture Organisation (FAO), and other organisations and parties to facilitate the identification, recognition, and reporting of OECMs (FAO 2019, 2022; IUCN-WCPA 2019; Garcia et al. 2021; ICES 2021; Jonas et al. 2023). These efforts aim to support the attainment of global biodiversity conservation targets alongside protected areas. A key milestone in this effort is Target 3 of the Kunming-Montreal Global Biodiversity Framework, commonly referred to as the "30 × 30" target, adopted in late 2022. This global initiative calls on governments worldwide to take coordinated and effective action to combat biodiversity loss by ensuring that at least 30% of terrestrial and marine areas are conserved by 2030 through ecologically representative, effectively managed, equitably governed, and well-connected networks of protected areas and OECMs (CBD 2022).

Although the OECM concept is widely recognised as a unique opportunity to support the 2030 biodiversity conservation agenda, concerns have been raised about its potential misuse by governments and economic sectors. There is a need to ensure that OECMs genuinely deliver the biodiversity benefits outlined in their definition (Alves-Pinto et al. 2021; Claudet et al. 2022; Lemieux et al. 2022). Proposed actions to prevent misuse and ensure that the OECM concept is effectively applied include prioritising area-based management approaches, forecasting expected conservation impacts on natural values and ecosystem services, and assessing avoided threats to biodiversity (Claudet et al. 2022). The provision of sector-specific, robust guidelines that would enable and facilitate the identification, recognition, support, reporting, and monitoring of OECMs across different governance types is essential (Dudley et al. 2018; Alves-Pinto et al. 2021; Gurney et al. 2021; Maini et al. 2023).

From their official definition in 2018 until recently, many countries, such as Canada, Algeria, Morocco, Colombia, the Philippines, and Japan, have made significant efforts to identify, recognise, and report OECMs to support the implementation of spatially explicit conservation targets. According to Protected Planet statistics, 17.55% of land and inland water ecosystems and 9.85% of coastal waters and the ocean are currently protected areas or OECMs. As of now, 6594 (6375 terrestrial and 219 marine) OECMs have been recognised across 15 countries, covering just 1.10% of the land and 0.24% of the global ocean (UNEP-WCMC and IUCN 2025). However, progress in OECM recognition remains uneven across regions. In the European Union (EU), only recently OECMs were recognised and officially reported to the World Database on OECMs (WD-OECM) by only one member state (BISE 2022; UNEP-WCMC and IUCN 2025). Sweden submitted 5365 OECMs in 2024 and successfully reported them in 2025, marking the first substantial national contribution to the WD-OECM from within the EU. Although the WD-OECMs figures have been recently judged as lacking sufficient documented evidence (Cook et al. 2025), they highlight the low progress made so far and the limited contribution of OECMs towards achieving global spatial conservation targets (Alves-Pinto et al. 2021; Cook 2024a; Jonas et al. 2024a). More importantly, they highlight the urgent need for accelerated action to ensure the recognition and official reporting of both protected areas and OECMs within the forthcoming five years, i.e., by 2030.

Research on OECMs has expanded in recent years, covering a range of important aspects of this emerging conservation concept. Studies have examined the progress in developing the OECM concept (Cook 2024a), OECMs typologies and the range of conservation strategies that could qualify as OECMs (Cook 2024b), the actual level of protection provided by OECMs (Jonas et al. 2024a), the challenges in OECM identification and recognition (Alves-Pinto et al. 2021; Jonas et al. 2024a), key knowledge gaps that need to be addressed to facilitate OECMs recognition (Cook 2024a, 2024b; Jonas et al. 2024a), concerns about OECMs misuse, misinterpretation and lack of credibility (Lemieux et al. 2019, 2022; Claudet et al. 2022; Cook et al. 2025) and the next critical steps required to demonstrate their conservation value (Gurney et al. 2021). Despite recent progress, OECMs remain comparatively understudied, poorly understood, and only marginally utilised at the global level, particularly when compared with protected areas. This highlights the need for further research to refine assessment approaches, strengthen methodological consistency, and develop practical tools to support effective OECM implementation. In this context, the main aim of this review is to systematically map and synthesise the global evidence base on potential OECMs, defined as areas proposed in the literature as potentially meeting the CBD OECM criteria. Rather than evaluating realised conservation outcomes of formally recognised OECMs (actual OECMs), this review focused on the processes, methodologies and evidence used to support potential OECMs identification and assessment, which underpin their potential contribution to global targets. To achieve this, a scoping review was conducted to (1) map the geographical distribution of studies identifying potential OECMs, (2) describe the characteristics of potential OECMs reported in the literature, including governance, sectors, realms, and conservation objectives, (3) document the methodologies and tools used to assess potential OECMs and their biodiversity contributions,(4) synthesise reported evidence on the spatial extent of potential OECMs and their effectiveness and (5) identify knowledge gaps and policy recommendations emerging from the literature. To our knowledge, this is the first systematic scoping review conducted on OECMs at a global scale.

## Methods

Scoping reviews are designed to provide a broad, structured overview of existing knowledge on a topic. They focus on systematically identifying, mapping, and summarising available evidence across diverse sources. This approach is especially useful in fields where concepts are emerging or where the literature is wide-ranging and unevenly distributed, as it helps clarify what is known, what remains uncertain, and where further research is needed (Arksey and O’Malley 2005; Levac et al. 2010; Peters et al. 2020a,b).

The methods for this scoping review were predetermined and published in an a priori protocol (Petza et al. 2023), which provides detailed information on the methodology used. Only minor protocol deviations occurred during review and data extraction, primarily related to adjustments in the data extraction tool and clarifications of the aim, the research question and the inclusion/exclusion criteria (Table S1). The scoping review adhered to the methodological framework outlined by Arksey and O'Malley (2005) and further developed by Levac et al. (2010) and the Joanna Briggs Institute (JBI) (Peters et al. 2020a,b). The Preferred Reporting for Systematic Reviews and Meta-Analyses extension for scoping reviews (PRISMA-ScR) checklist (Tricco et al. 2018) guided both the protocol development and the final scoping review paper (Table S2).

### Review question

The central research question guiding this scoping review was: What is the current knowledge on how potential OECMs are identified, assessed, and reported to contribute to biodiversity conservation targets? To address this, the review explored the following sub-questions: (1) Where are potential OECMs identified globally? (2) What types of potential OECMs are identified (governance, sector, realm, objectives)? (3) What assessment approaches and tools are used? (4) What spatial extent of potential OECMs is documented? (5) What are the reported outcomes (i.e., effectiveness, gaps, and policy recommendations)?

### Inclusion/exclusion criteria

The inclusion criteria for this scoping review, which define the sources considered for inclusion, were developed following the "Participants, Concept, and Context (PCC)" mnemonic (Peters et al. 2020a,b; Table 1). The review considered potential ΟECMs (i.e., sites with OECM-like characteristics) established by any sector, such as transport, offshore energy, fisheries, aquaculture, maritime, tourism, defence, and archaeological heritage. We considered potential OECMs with three types of conservation objectives: primary when sites meet all elements of the IUCN definition of a protected area, but lack official designation, secondary when biodiversity outcomes are a secondary management objective of the site, and ancillary when in situ conservation results as a by-product of site management (IUCN-WCPA 2019). These areas may be governed by various entities, including governments (at various levels), individuals, organisations or companies, indigenous peoples and local communities, or through shared governance involving multiple rights holders and stakeholders. Studies that refer to actual OECMs, i.e., those identified, recognised and reported in the WD-OECM, were not included in the scoping review. The review focused on how potential OECMs are assessed and identified, as well as how their contribution to spatial conservation targets is addressed in existing scientific literature. All studies that assessed and proposed potential OECMs, proposed OECM screening tools or discussed OECM assessments were included. The various methodologies and metrics applied to evaluate their effectiveness in delivering biodiversity conservation outcomes and their contribution to spatial conservation targets were reviewed. The scoping review considered studies across the terrestrial, freshwater, and marine realms worldwide (Table 1).

**Table 1:** Inclusion and exclusion criteria for the Scoping Review in correspondence with the "Participants, Concept and Context, PCC" mnemonic and evidence types and sources

	Inclusion criteria	Exclusion criteria
PARTICIPANTS *Potential other effective area-based conservation measures (OECMs)*	Potential OECMs governed under a range of governance types i.e., by governments (at various levels), private individuals, organisations or companies, indigenous peoples and local communities (IPLCs) and shared governance (i.e., governance by various rights holders and stakeholders together) Potential OECMs established by all sectors (e.g., transport, offshore energy, fisheries, aquaculture, maritime, tourism, defence, archaeological heritages, etc.) Potential OECMs with primary, secondary or ancillary conservation objectives	OECMs which are referred to by the study as actual OECMs, i.e., which have been identified, recognised and reported at the World Database on OECMs (WD-OECM)
CONCEPT *Assessing potential OECMs*	All studies that assess or propose potential OECMs. All studies that propose potential OECMs screening tools All types of methodologies and metrics applied to assess the effectiveness of potential OECMs to deliver biodiversity conservation outcomes and contribute to spatial conservation targets	–
CONTEXT *Global terrestrial, freshwater and marine realm*	Studies in: Terrestrial, freshwater and marine realms Globally	–
EVIDENCE TYPES & SOURCES	Peer-review literature Grey literature All years of publication All publication stages, subject areas, and source types Experimental and observational studies Studies published in languages competent to the researchers' team (e.g., English, French, German, Greek, Italian, Spanish, etc.)	Evidence Synthesis such as Systematic, Scoping, Rapid, and Narrative Reviews

### Search strategy and types of sources

This scoping review encompassed both scientific and grey literature. The bibliographic search was conducted on multiple online platforms, i.e., Scopus, Web of Science—Core Collection, and Google Scholar. Eligible studies were also sought in other sources (Table S3), such as pre-print archives, thesis/dissertation archives, organisational libraries and websites, document/data repositories, web-based search engines, reference lists of included documents, and expert suggestions. A combination of keywords was used in the search, tailored to meet the search specifications of each database and source (Table S3).

Given that OECMs represent a relatively new and evolving conservation concept, the search strategy was designed to maximise sensitivity and minimise the risk of missing relevant evidence. The search string incorporated all known terminology used to refer to OECMs, including long-form expressions, hyphenated and non-hyphenated variants, and historical acronyms (e.g., ‘other effective area-based conservation measure’, ‘other conservation measure’, ‘OECM’, ‘OEABCM’). Broader conservation terms or keywords relating to other area-based measures were intentionally not included, as the purpose of this review was to capture studies that explicitly address OECMs to understand how the concept itself is being developed, interpreted, and assessed in the literature. As terminology in this field continues to stabilise, a broad yet concept-focused string ensured coverage of all relevant references without introducing large volumes of studies outside the review’s scope. To further strengthen comprehensiveness, we conducted backward citation chasing (screening reference lists of all included papers) and forward citation chasing (identifying newer citing papers through Scopus, Web of Science, and Google Scholar), alongside extensive searches of grey literature sources. This multi-step strategy ensured coverage beyond formal indexing systems and captured studies that refer to OECMs using diverse phrasings, while remaining aligned with the review’s focus on the evolution of the OECM concept itself.

No restrictions on publication year, publication stage (final or in press), subject area, or source type were applied. All document types, except evidence synthesis, such as systematic, scoping, rapid, and narrative reviews, were considered. Studies written in English, French, German, Greek, Italian, and Spanish were included, in alignment with the language competence of the authors.

Following the search process, all identified citations were uploaded to the Covidence systematic review software (Covidence 2025). The document selection process was performed using a team-based screening approach, as recommended by Levac et al. (2010).

### Data extraction, synthesis and presentation

Data extraction from each selected document was performed using a data extraction tool, specifically a charting table aligned with the objectives and research questions of the scoping review (Table S4). The extracted data included specific details on the studies (e.g., authors, title, year of publication), the participants (e.g., designation name, sector, designation rationale, conservation objective), the context (e.g., continent, country, realm), the concept, and study methods characteristics (e.g., type of research, data collection and analysis methods, metrics), and key findings (e.g., effectiveness, candidate status, knowledge gaps as reported by the authors of each included study not interpreted by this review as formal judgements on sites’ eligibility or recognition) relevant to the review objective, structured into 38 fields (Table S4). Data were extracted for the methods used to collect (how information was obtained) and analyse (how collected information was synthesised to evaluate potential OECMs) data. Data synthesis refers to cases in which authors qualitatively integrated existing information without applying formal analytical techniques. Assessment refers to the overall evaluation of potential OECMs reported by study authors, while analysis refers specifically to the analytical techniques used to process or interpret data as part of that assessment. Methodological analyses, metrics, effectiveness and candidate status were recorded only for case studies that explicitly assessed potential OECMs. To ensure consistency and facilitate collaboration among reviewers, the data extraction tool was integrated into Covidence.

The synthesised evidence is presented in alignment with the review objective and questions, which is a core requirement of the JBI methodology for scoping reviews (Peters et al. 2020a,b). The collected data were analysed by applying descriptive statistical methods. Statistical analysis was performed in R version 4.4.2, using the stats package (R Core Team 2025). The summarised data were presented through a combination of graphical and tabular formats, utilising appropriate software packages and tools (Microsoft Excel, Flourish Studio, Datawrapper). Graphical representations were used to visually display relevant information and trends, and to convey patterns, relationships, and key findings. Additionally, the results were presented in a narrative summary, providing a coherent and comprehensive synthesis of the findings. This summary outlines how the results align with the review's objective and research questions, emphasising the key themes, trends, and patterns identified in the included studies.

## Results

### Scoping review workflow and dataset

**Fig. 1:**
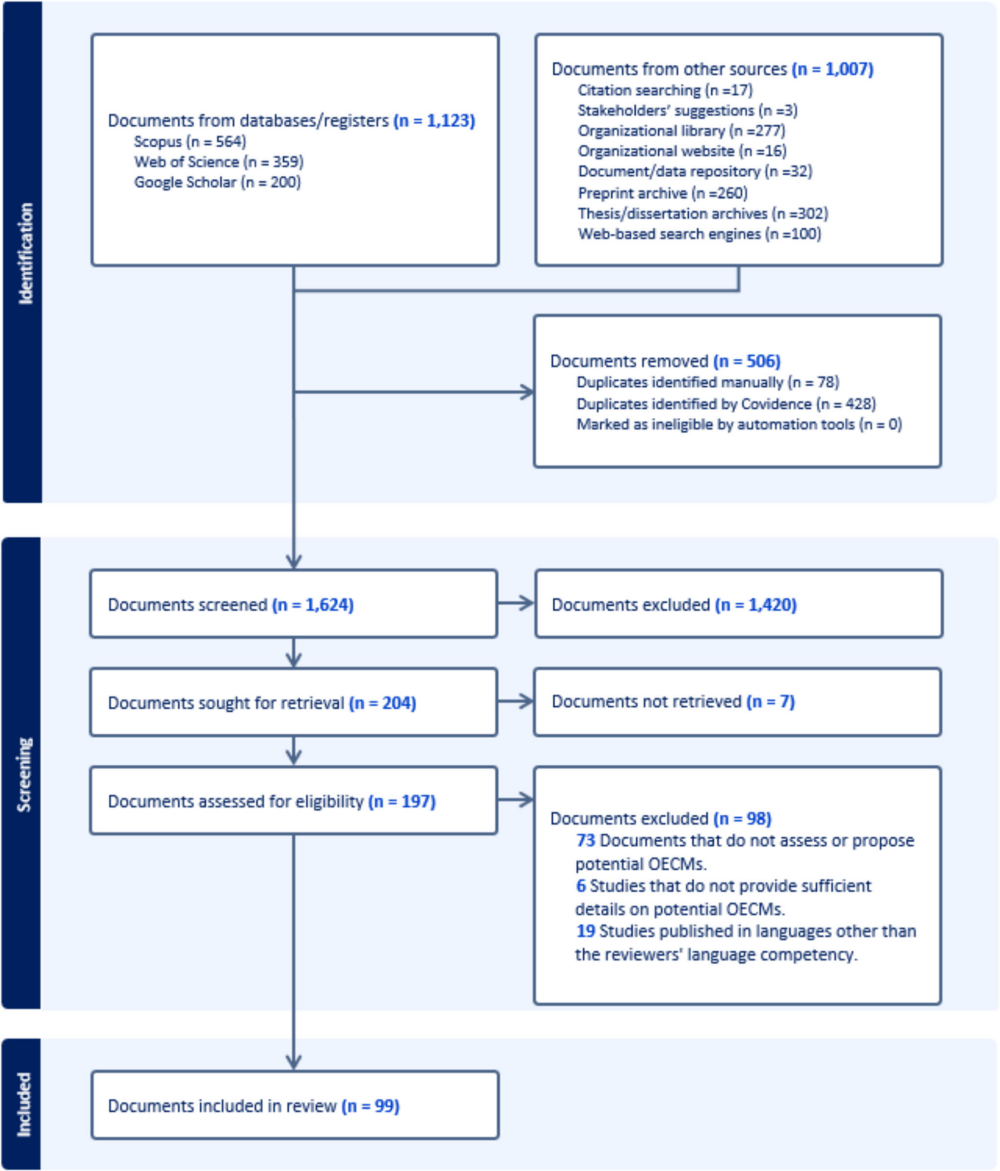
Preferred Reporting Items for Systematic Reviews and Meta-Analyses (PRISMA) flow diagram for the systematic search and review (as proposed by Page et al. (2021))

Through the initial search in databases, 1123 documents were identified, and 1007 additional documents came from other sources (Fig. 1). After removing 506 duplicates, 1624 documents were screened for eligibility based on titles and abstracts. Of these, 1420 were excluded as irrelevant, leaving 204 studies for full-text review. At this stage, seven documents could not be retrieved, and 98 were excluded, primarily because they did not propose or assess potential OECMs (*n* = 73), lacked adequate information (*n* = 6), or were written in languages outside the reviewers' language competency *(n* = 19) (Table S5). Consequently, 99 documents met the inclusion criteria (Fig. 1).

The 73 excluded documents referring to potential OECMs only superficially mentioned the concept briefly without presenting sites, assessments, case studies, or relevant methods, and therefore did not meet the eligibility criteria focused on explicit identification or assessment of potential OECMs. From the 99 included documents, 694 case studies were identified, recorded, and reviewed. Following data extraction, the information was compiled into a database with one row per document and additional rows for each case study. The majority of the documents included a single case study (*n* = 39), while others contained from 2 up to 53 case studies. The final database comprised 38 columns (each representing a specific data extraction field/characteristic) and 755 rows (Table S6; see the “ReadMe” sheet).

### Literature characteristics

**Fig. 2:**
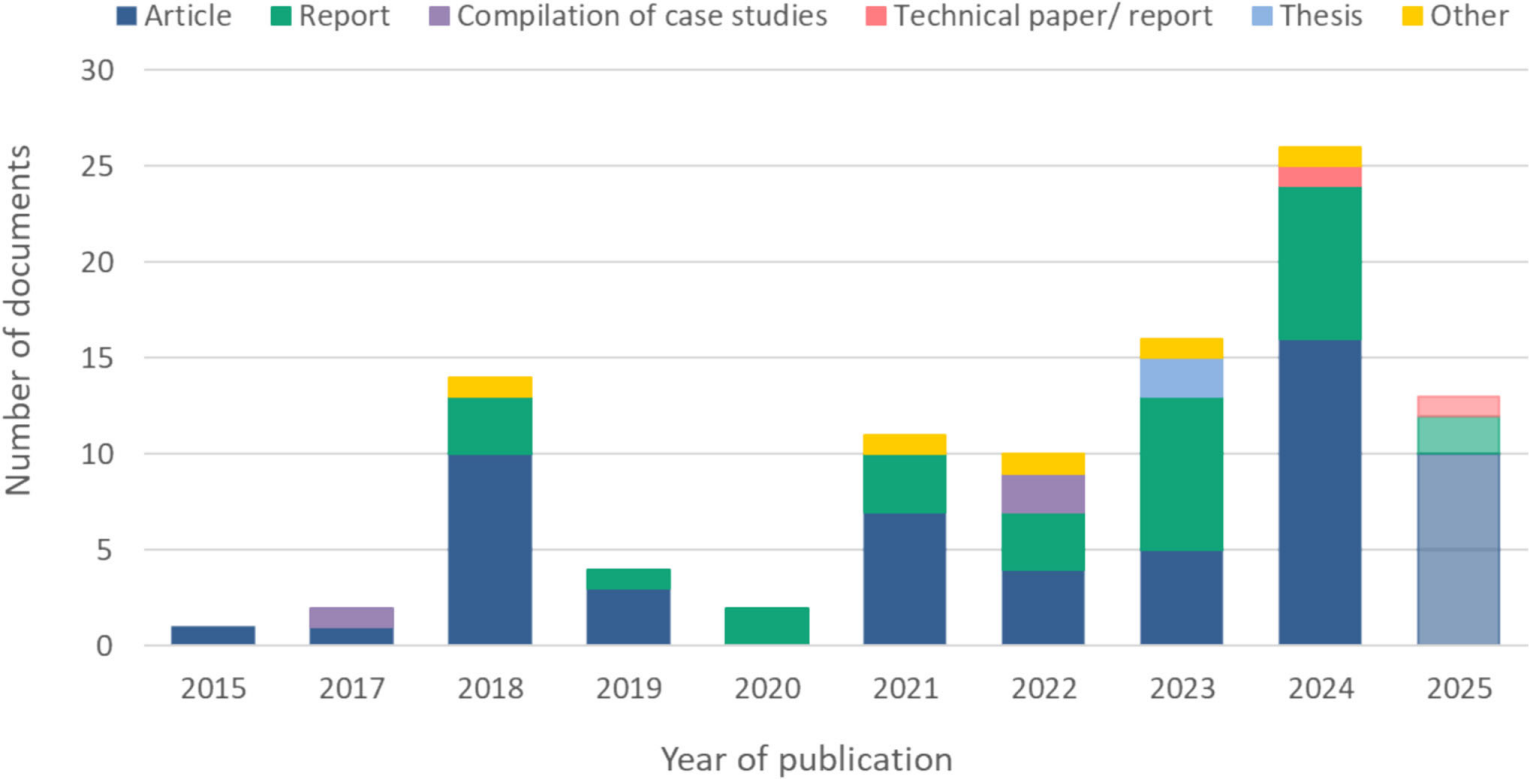
The number of documents included in the Scoping Review (n = 99) by literature type and year of publication (from 2015 to 2025). As the search for the documents was conducted in September 2025, it did not cover the whole year, and thus the bar corresponding to 2025 is displayed with transparent colours. Other = booklet (n = 1; 2018), conference paper (n = 1; 2022), meeting summary (n = 1; 2023), preprint (n = 1; 2024) and short communication (n = 1; 2021) (Fields #13 & 6 of the database)

Of the 99 documents included in the scoping review, 57 were peer-reviewed (56 articles, and one short communication), and 42 were grey-literature documents (30 reports, three compilations of case studies, two technical papers/reports, two theses, one article, one conference book of abstracts, one preprint, one meeting summary, and one booklet). The studies were published between 2015 and 2025 (Fig. 2), with all documents written in English except for two in Spanish and one in German. The largest share of the peer-reviewed documents were published in policy-oriented academic journals (*n* = 19), and conservation-oriented journals (*n* = 16). Grey literature documents were primarily published by international and intergovernmental organisations such as IUCN, FAO, ICES, NGOs, including WWF and EBCD, and by universities, institutions and private initiatives (Table S6).

### Case studies overview

The current scoping review primarily considered case studies that either identify (*n* = 436) or identify and assess (*n* = 257) potential OECMs. A small number developed and proposed screening tools (*n* = 13) or discussed OECM assessments (*n* = 1) (Figure S1A).

Among the tools proposed in the reviewed documents were:

- a decision-screening tool developed by the Canadian Council on Ecological Areas (CCEA) to assess sites for inclusion in Canada's Aichi Target 11 commitment employing a consensus-based approach to operationalise OECMs (MacKinnon et al. 2015; CCEA 2018),
- an operational framework integrating OECMs identification into marine spatial planning (Shabtay et al. 2019),
- a multi-criteria decision analysis tool based on expert judgment to evaluate area-based fisheries management measures under the OECMs concept (Petza et al. 2019) and
- a three-step, eight-criteria framework for assessing whether an area qualifies as an OECM under the CBD definition, including screening, consent, and full evaluation of governance, biodiversity values and long-term conservation outcomes (Jonas et al. 2023).

During screening and data extraction, several sector- and region-specific guidance documents relevant to OECMs were identified, although this was beyond the original scope of this review. These include the IUCN Guidelines for recognising and reporting OECMs (IUCN-WCPA 2019), the FAO Handbook for identifying, evaluating and reporting OECMs in marine fisheries (FAO 2022), the IUCN Guidance on OECMs (Jonas et al. 2024a, b), the Briefing Note on OECMs for the conservation and wise use of wetlands (Convention on Wetlands 2025), the Recommendations for a future implementation of the OECMs concept in France (Comité français de l’UICN 2022) and the Australian National OECMs Framework (Commonwealth of Australia 2024). Given their potential value for practitioners and policymakers, these documents are compiled in Supplementary Table S7 to provide an overview of existing guidance.

In the largest proportion of case studies assessing potential OECMs, the criterion for sustained and effective in situ biodiversity conservation (Criterion C) was addressed (232 studies). Many case studies also examined the site’s protection status (Criterion A; *n* = 229) and governance and management aspects (Criterion B; *n* = 228). Fewer studies considered associated ecosystem functions, services and cultural, spiritual, socio-economic and other locally relevant values (Criterion D; *n* = 104) (Figure S1B).

### Geographic and spatial distribution of case studies

**Fig. 3:**
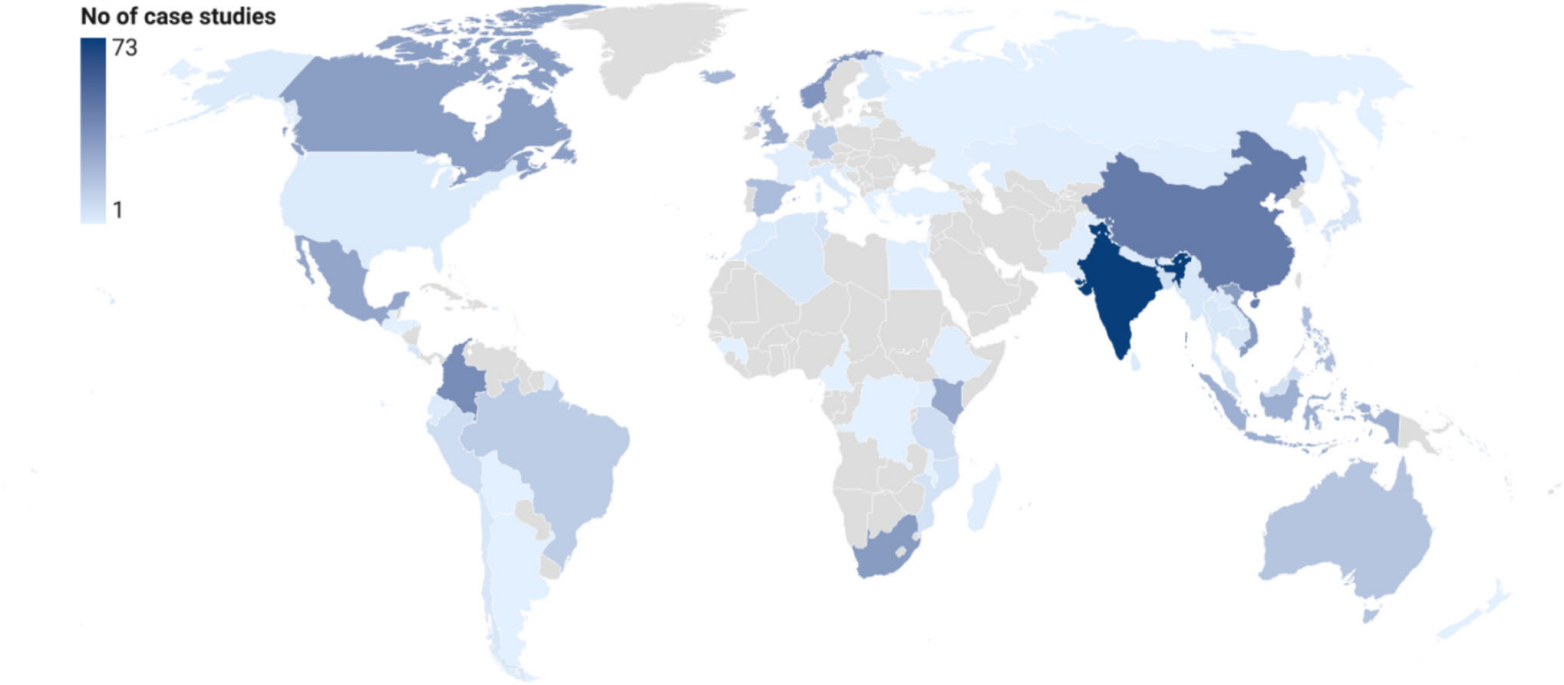
Global geographic distribution (choropleth map) of the number of case studies included in the Scoping Review by country (Field #16 of the database). The grey colour indicates countries with no case study

Most studies were conducted at the national level (*n* = 35), followed by sub-national (*n* = 32) and multinational (*n* = 17) scales. Eight documents included case studies across multiple spatial scales, while in five, the scale could not be determined. Only two studies were conducted at the global level (Figure S3). These examined socio-ecological production landscapes (SEPL) using the Satoyama Index on a global level (Natori and Hino 2021) and the development and testing of indicators to assess OECM eligibility, using global sample of sites (Cook et al. 2024). The case studies were geographically widespread, covering all continents except for Antarctica. Approximately one third originated from Asia (38%), followed by Europe (21%), Africa (14%), North America (11%), and South America (10%. Oceania/Australia accounted for 6% (Figure S2). Case studies were reported from 71 countries. The highest number were in India (*n* = 73), China (*n* = 46) and Colombia (*n* = 39), followed by Norway (*n* = 36), South Africa and Vietnam (*n* = 33 each), and Canada (*n* = 32) (Fig. 3).

### Characteristics of potential OECMs

The 694 reviewed case studies proposed approximately 237 000 sites as potential OECMs. Reported site sizes varied by more than six orders of magnitude, ranging from 0.0005 to 1 440 000 km^2^, with a median of 26 km^2^. This wide range reflects the diversity of area-based measures considered under the OECM concept by the literature, spanning very small, site-specific interventions to large, landscape- or seascape-scale management areas. However, information on spatial extent was not reported consistently across studies, limiting more detailed comparative analyses of size distributions across sectors, realms, or governance types.

**Fig. 4:**
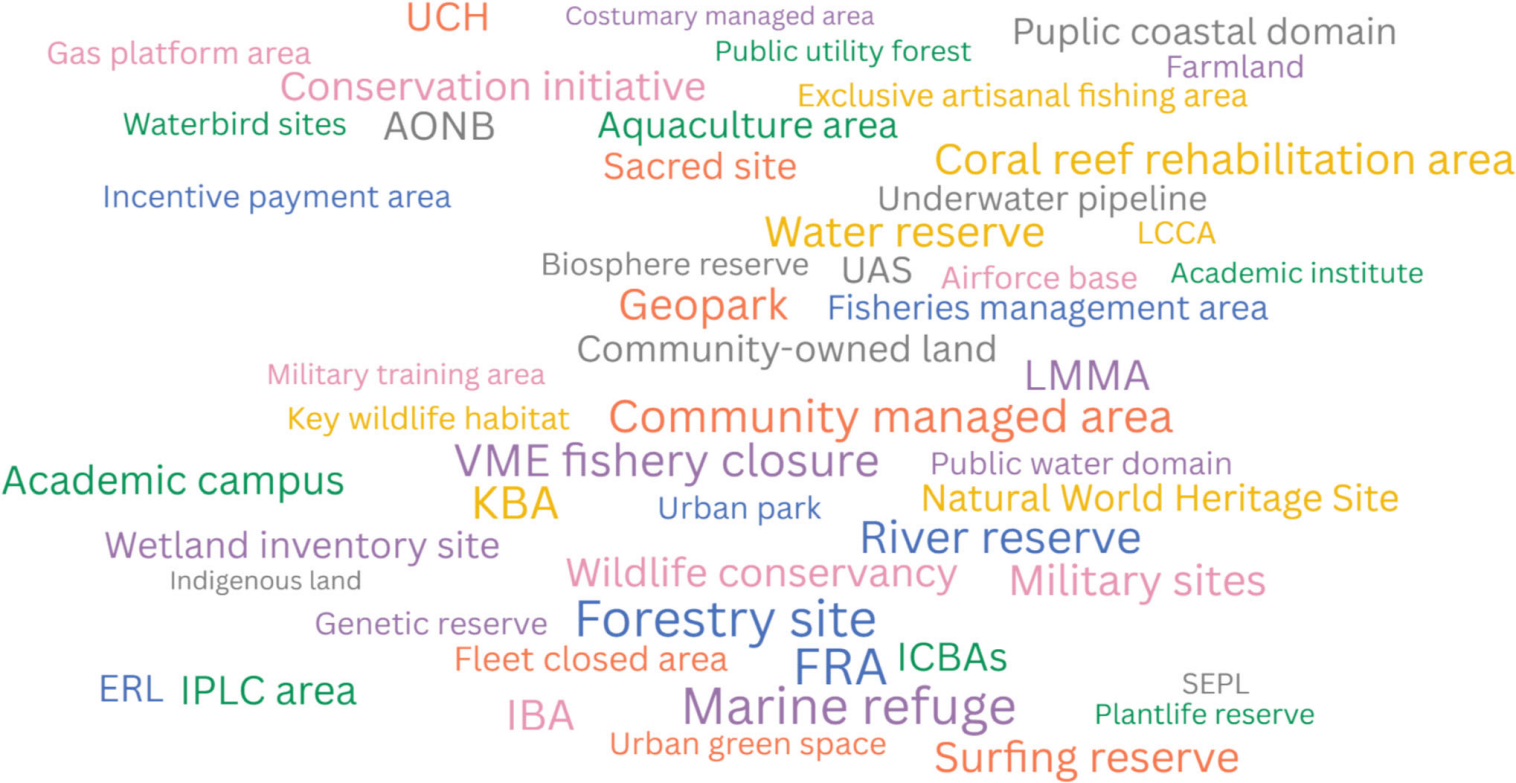
Word cloud of the different types of potential OECMs based on their designation name as reported in the reviewed documents. AONB = Area of Outstanding Natural Beauty, ERL = Ecological Red Line, FRA = Fisheries Restricted Area, IBA = Important Bird Area, ICBA = Important Coastal Biodiversity Area, IPLC = Indigenous Peoples and Local Communities areas, LCCA = Locally Coral Conservation Area, LMMA = Locally Managed Marine Area, SEPL = Socio-ecological production landscape, UAS = Unique Agricultural System, UCH = Underwater Cultural Heritage, VME = Vulnerable Marine Ecosystem (Field #20 of the database)

**Fig. 5:**
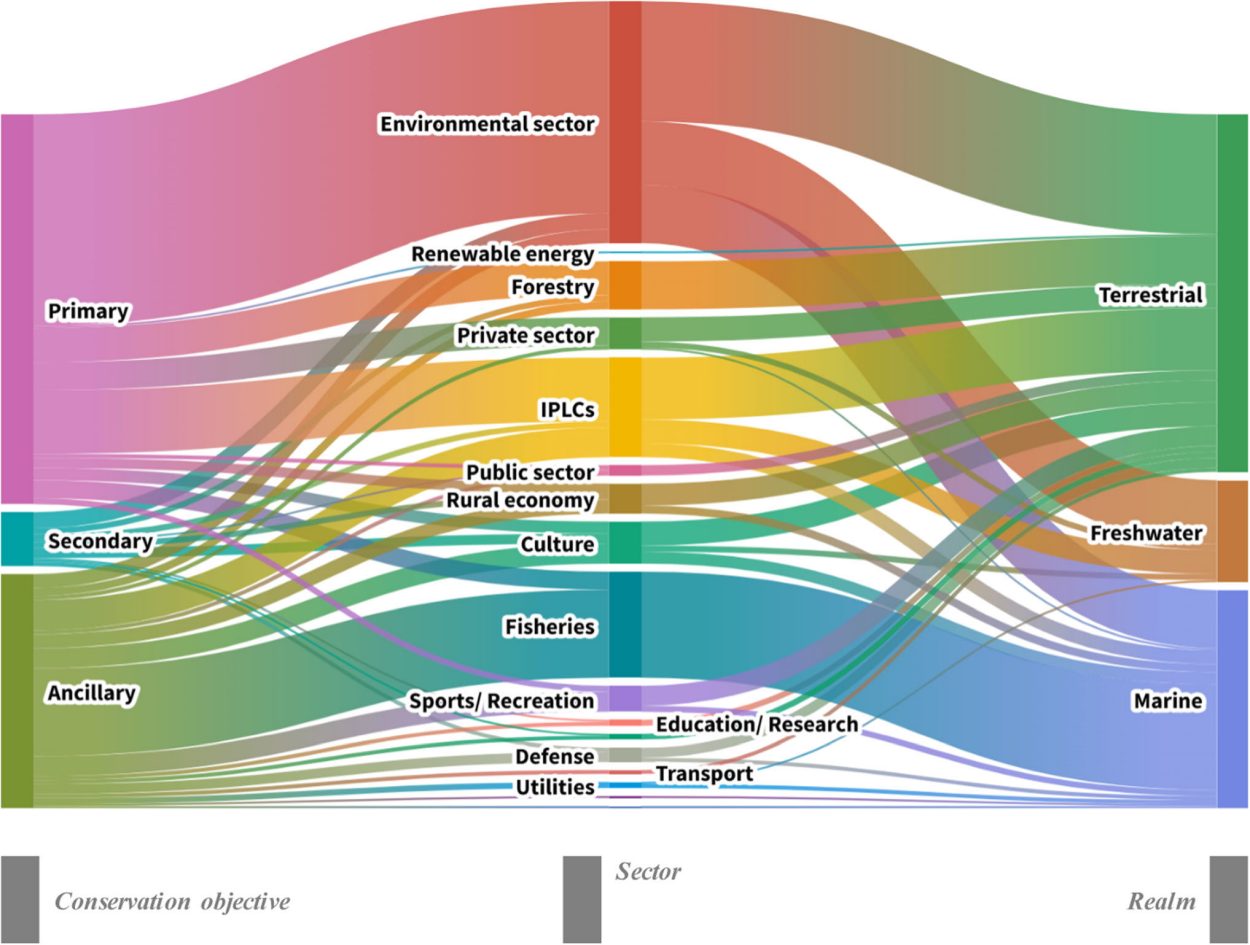
Sankey diagram representing the frequency in the combination of i) conservation objectives of the potential OECMs, ii) the OECMs sector and iii) the realm. The width of the nodes and lines is proportional to the flow quantity (i.e., the number of potential OECMs) (Fields #22, 20 & 17 of the database). The sector classification is based on the International Labour Organisation classification and adequately adjusted to align with the needs of the current study. IPLCs: Indigenous Peoples and Local Communities

Potential OECMs spanned terrestrial (39%), marine (32%), and freshwater (15%) realms, with 9% being cross-realm (Figure S4). A wide range of potential OECM types was identified, including marine refuges, community-managed areas, natural parks, fisheries management areas, aquaculture areas, military sites, underwater cultural heritage sites, river reserves, important bird areas, key biodiversity areas, vulnerable marine ecosystem closures and more (Fig. 4). Primary conservation objectives were reported in 43% of case studies, with 30% having ancillary and 10% secondary conservation objectives, based on their designation rationale (for 17% the type of conservation was not reported; Fig. 5, Figure S5). These identified potential OECMs spanned a wide range of sectors. While the Environmental sector played a leading role, other key contributors included Fisheries, IPLCs, Private sector (privately owned areas), Forestry, Culture, Rural Economy, Transport, Defence, Sports and Recreation, Education and Research, Utilities (water, gas, electricity), Public sector, and Tourism. In addition, a significant number of multi-sectoral case studies were represented, with the Environment, IPLCs, Forestry, Private sector, Tourism, Fisheries, and Culture being the most involved (Fig. 5, Figure S6). Potential OECMs proposed by the Environmental sector predominantly had primary conservation objectives and covered all three realms (mainly terrestrial and marine). Potential OECMs associated with the Fisheries sector mainly had ancillary or secondary conservation objectives and were exclusively marine. Those associated with the IPLCs encompassed all three objective types (most commonly primary) and occurred across all three realms (mainly terrestrial and freshwater). Potential OECMs with ancillary conservation objectives were identified across all sectors (Fig. 5).

### Methodological aspects

**Fig. 6:**
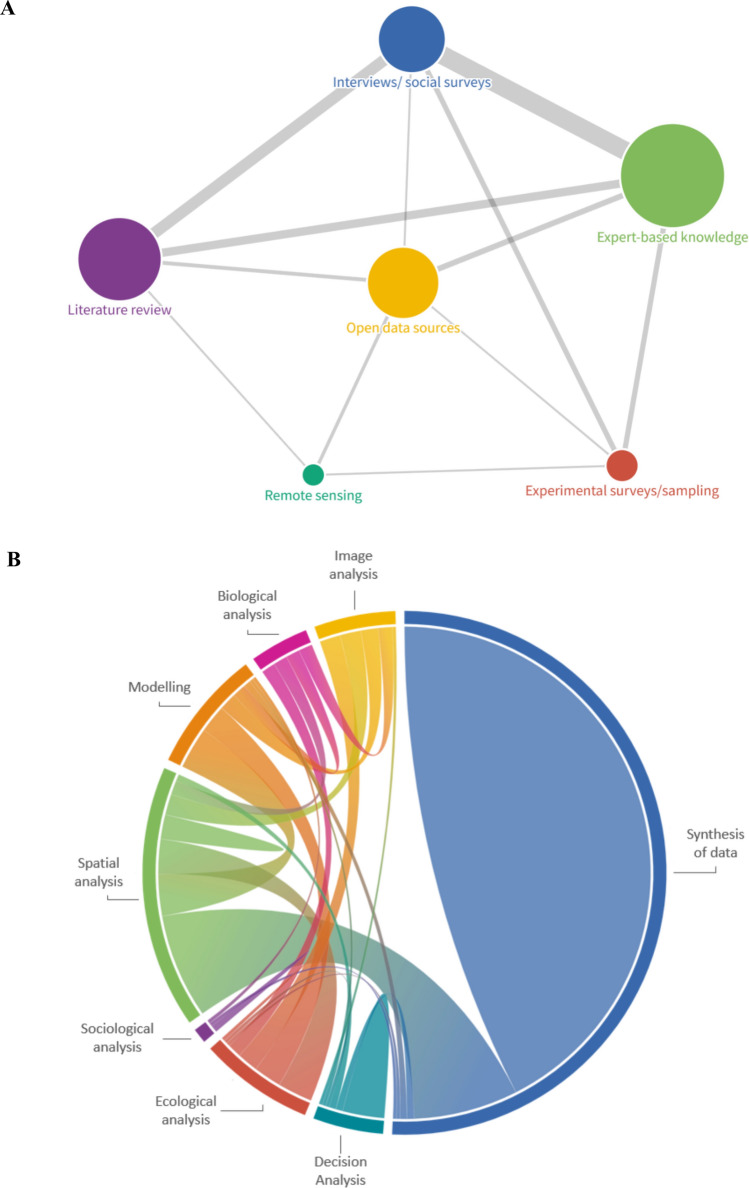
**A** Network graph representing the connections between the various data collection methods used to assess potential Other Effective area-based Conservation Measures (OECMs) in the case studies included in the Scoping Review. The size of the links is proportional to the importance of the connection, and the size of the circles is proportional to the number of case studies that applied each data collection method (Field #28 of the database). **B** Chord diagram representing the connections between the various data analysis methods used to assess potential Other Effective area-based Conservation Measures (OECMs) by the case studies included in the Scoping Review. The arc size is proportional to the importance of the flow (i.e., the number of case studies that applied each data analysis method). (Field #29 of the database)

The methodological results presented below refer exclusively to case studies that explicitly assessed potential OECMs (*n* = 257), rather than those that solely identified potential OECMs. The majority of case studies applied qualitative approaches to assess potential OECMs (78%; Figure S7). Expert-based knowledge was the predominant data collection method (*n* = 160), followed by literature reviews (*n* = 103), open data sources (*n* = 76), interviews and social surveys (*n *= 67), experimental surveys and sampling (*n* = 16), and remote sensing (*n* = 8). A single data collection method was used in 107 case studies, while others combined multiple methods (127 used two, 38 used three and two used four). The most frequent combinations paired expert-based knowledge with interviews/surveys, literature review, or experimental surveys/sampling. Interviews/social surveys were also commonly combined with experimental surveys/sampling. Open data sources were frequently used with expert-based knowledge and literature reviews (Fig. 6A and Figure S8). Overall, mixed-method designs were frequent, with expert knowledge and interviews serving as core methods, literature reviews providing broad support, and remote sensing and experimental sampling used more selectively.

Various data analysis methods for assessing potential OECMs were identified in the reviewed studies. The vast majority (*n* = 220) performed data synthesis alone (i.e., narrative or descriptive integration of information without analysis). Among studies that applied analytical methods, spatial analysis (e.g., GIS-based mapping spatial overlays, area calculations; *n* = 50), decision analysis (e.g., multi-criteria decision analysis or structured expert judgement frameworks; *n* = 16), modelling (e.g., ecological, spatial, or scenario-based models; *n* = 12), ecological analysis (e.g., analysis of species composition, habitat condition, or ecosystem attributes; *n* = 11), image analysis (e.g., interpretation of satellite or aerial imagery; *n* = 6), biological analysis (e.g., species-level or population-based analyses; *n* = 4) and sociological analysis (e.g., analysis of social survey or interview data; *n* = 3) were reported (Fig. 6B; Figure S9; details in Table S6, Field #26).

**Fig. 7:**
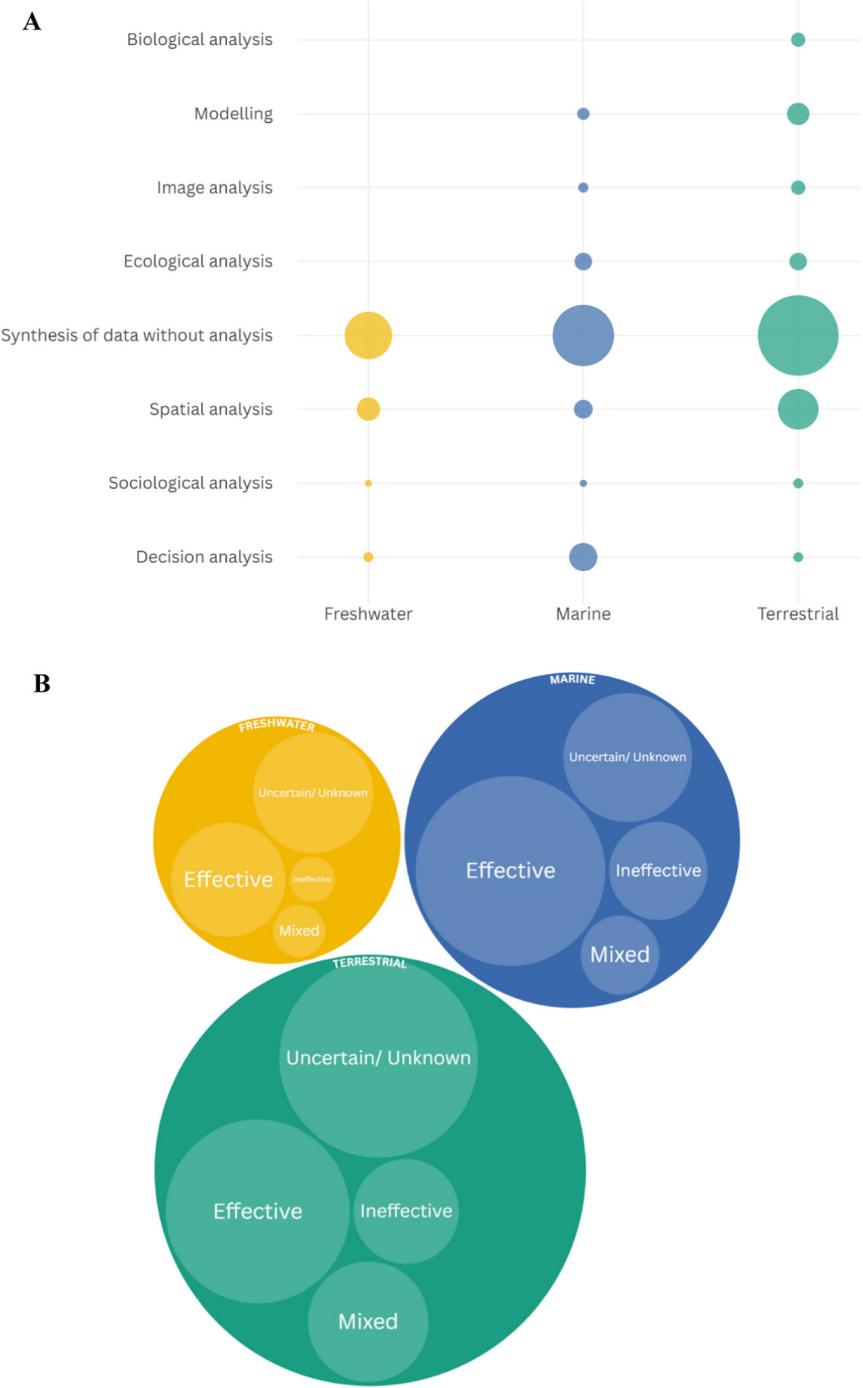
**A** Scatterplot (bubble chart) representing the relationship between the realm (terrestrial, marine and freshwater) and the various data analysis methods (synthesis of data without analysis, decision analysis, modelling, spatial analysis, and other, i.e., ecological analysis, biological analysis, image analysis and sociological analysis) used to assess potential Other Effective area-based Conservation Measures (OECMs) by the case studies included in the Scoping Review. The bubble size is proportional to the importance of the relationship (i.e., the number of case studies by data analysis method and realm) (Fields #17 and 29 of the database). **B** Hierarchy (packed cycles) chart representing the relationship between the realm (terrestrial, marine and freshwater) and the effectiveness (effective, ineffective, mixed, uncertain/unknown) of the potential Other Effective area-based Conservation Measures (OECMs) case studies assessed, as reported by the authors of the studies included in the Scoping review. The cycle size is proportional to the importance of the relationship (i.e., the number of case studies by realm and effectiveness). (Fields #17 and 32 of the database)

In 209 case studies, a single analysis method was used. Except for data synthesis, which dominated as a standalone approach, all other data analysis methods were frequently combined to assess potential OECMs. Spatial analysis showed the strongest integration, often paired with data synthesis, ecological analysis and modelling. Ecological analysis was also frequently paired with spatial analysis or modelling, and modelling also linked with biological and image analysis. Modelling displayed broad connections across several approaches (Fig. 6B). Synthesis without additional analysis was the most prevalent method across all realms, particularly in terrestrial studies. Several analysis types were used only in specific realms. Freshwater studies included four methodological categories, i.e., decision, sociological, spatial, and synthesis, whereas marine and terrestrial studies displayed a broader range of approaches. Spatial analysis occurred in all realms but was especially prominent in terrestrial research. Modelling, ecological, image, and biological analyses were rare or absent in freshwater studies and less frequent in marine studies (Fig. 7A). The distribution of analysis types differed among realms (chi-square test; χ^2^ = 43.04, *p*-value < 0.001, d*f* = 14), indicating non-uniform patterns. These findings suggest a higher reliance on unsupplemented data synthesis in terrestrial research, whereas analytical methods such as decision and spatial analyses remain underutilised across all realms, possibly reflecting differences in data availability, research focus, or methodological preferences.

The reviewed studies employed a range of metrics to assess potential OECMs, including species diversity, total abundance, community composition, pressure-state-response scores from in situ monitoring, the Satoyama Index, vegetation conversion and regrowth, scores reflecting governance principles (e.g., rights and actors, participation, transparency, accountability), Shannon diversity equitability indices, connectivity level, energetic cost among others. However, 70% of case studies (180/257) did not report using any metrics (Table S6, Field #30). More than half of the case studies assessing potential OECMs (176/257 case studies; 68%) evaluated them against the CBD criteria. Notably, the majority of the case studies that did not apply the CBD criteria (59/81 case studies; 73%) were published before the adoption of CBD Decision 14/8 in 2018.

### Potential OECM effectiveness and candidate status

In this review, reported effectiveness reflects the authors’ own assessments of whether potential OECMs are deemed effective for the aspects or criteria assessed, and not an independent evaluation by the review team. Effectiveness classifications (effective, ineffective, mixed, uncertain/unknown) therefore capture study-specific judgments expressed by the authors of the reviewed studies, based on particular indicators, criteria, or management objectives. On this basis, over 40% of case studies assessing potential OECMs considered them effective for the criteria evaluated. About one-third reported uncertainty or insufficient knowledge regarding their effectiveness, while 13% indicated mixed effectiveness, with some metrics indicating positive outcomes and others not. Only 13% classified potential OECMs as ineffective (Figure S10). Patterns in reported OECM effectiveness differed among realms (chi-square test; *χ*^2^ = 13.91, *p*-value = 0.031, df = 6). Many studies classified potential OECMs as having uncertain or unknown effectiveness, particularly in terrestrial systems. Studies commonly reported potential OECMs as effective in terrestrial and freshwater contexts, whereas ineffective or mixed outcomes were rare. In marine environments, a more even distribution of outcomes was reported. Overall, the distribution of author-reported assessments of effectiveness indicates substantial variation across realms, with persistent uncertainty, especially in terrestrial systems, and more heterogeneous evaluations in marine studies (Fig. 7B).

**Fig. 8:**
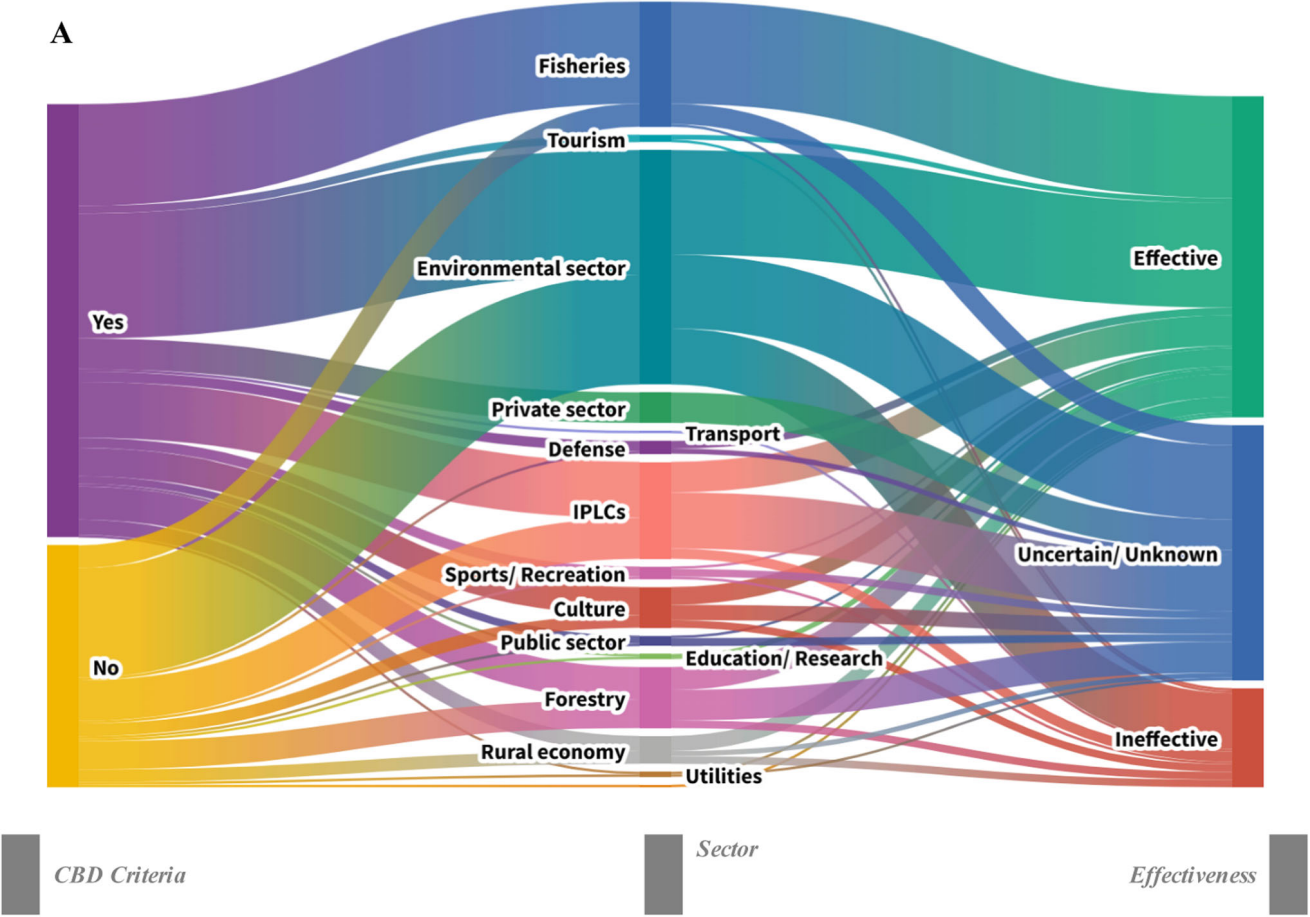

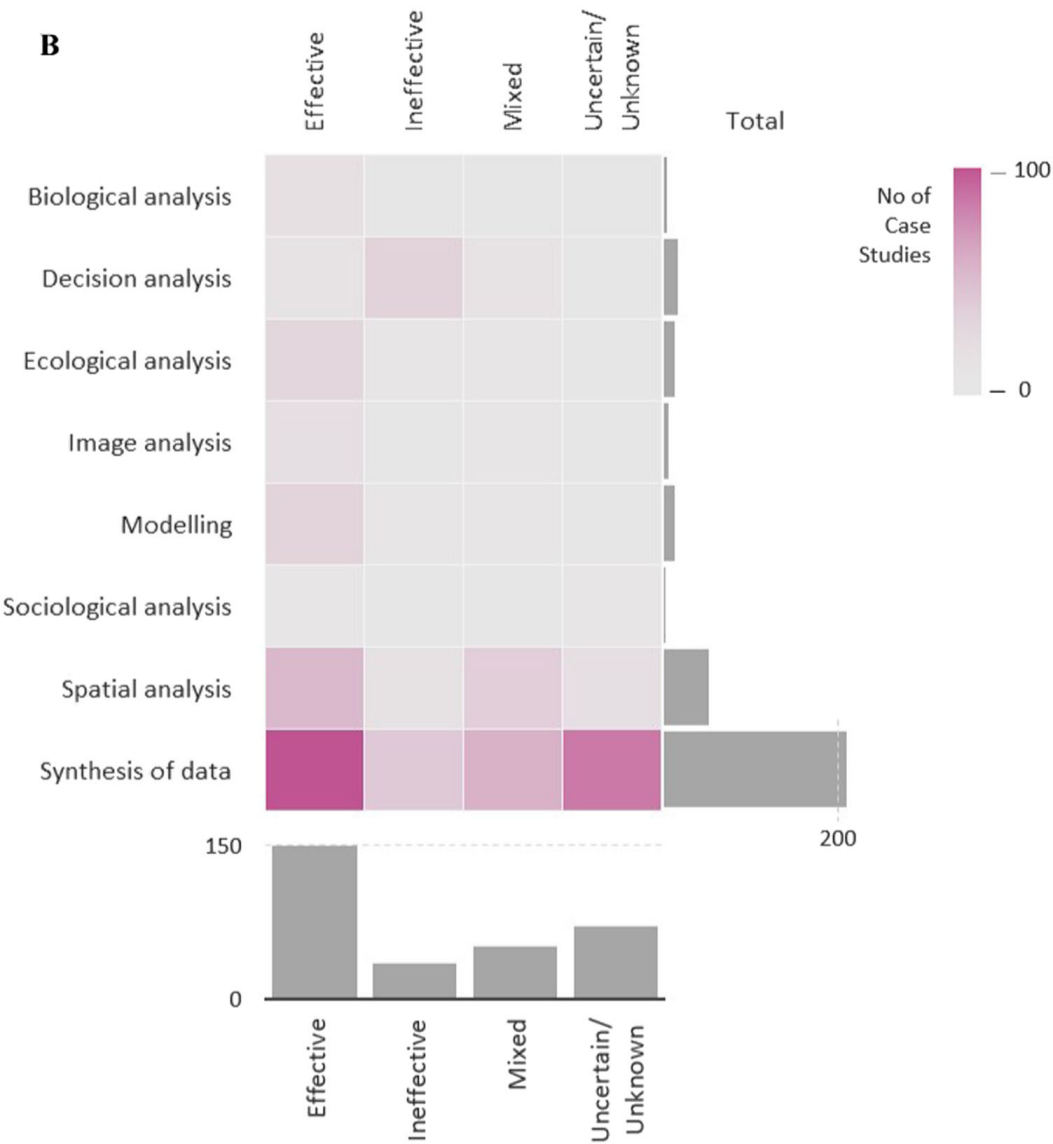
**A** Sankey diagram representing the frequency in the combination of i) potential Other Effective area-based Conservation Measures (OECMs) case studies effectiveness as reported by the authors of the studies included in the Scoping review, i.e., effective, ineffective, uncertain/unknown ii) the OECMs sector, i.e., Defence, Fisheries, Environmental sector, Rural economy, Tourism, Education/research, Sports/Recreation, Culture, Indigenous Peoples and Local Communities (IPLCs), Tourism, Private sector, Transport, Public sector, Forestry, Utilities and iii) use (Yes) or not use (No) of the CBD OECMs Criteria for the potential OECMs assessments (Fields #32, 20 & 26 of the database). The sector classification is based on the International Labour Organisation classification and adequately adjusted to align with the needs of the current study. **B** Heat map showing the number of potential Other Effective area-based Conservation Measures (OECMs) case studies by effectiveness category (i.e., effective, ineffective, mixed, uncertain/unknown), as reported by the authors of the studies included in the Scoping Review, across different data analysis methods. Darker shading indicates a higher number of case studies. Marginal bars show the total number of case studies for each effectiveness category and analysis method

Potential OECMS from the Environmental, Fisheries and IPLCs sectors were most often judged effective (Fig. 8A). In contrast, several other sectors, notably Forestry, Rural economy, and parts of the Public and Private sectors, were more frequently linked to uncertain or unknown effectiveness, with fewer ineffective outcomes. Tourism, Education/Research, and Transport showed mixed patterns. Case studies applying CBD criteria aligned more often with effective assessments, whereas those not applying them were more frequently associated with uncertain or ineffective outcomes. The distribution of effectiveness categories differed among analysis types (chi-square test; *χ*^2^ = 108.13, *p* < 0.001, d*f* = 21). Data synthesis dominated across all effectiveness categories, particularly for cases classified as effective and uncertain/unknown. Spatial analysis was more frequently associated with effective and mixed outcomes compared to other analytical approaches, whereas decision analysis showed a higher relative association with ineffective outcomes (Fig. 8B). Approximately half of the case studies explicitly proposed the assessed areas as candidate OECMs. In the reviewed studies, whether a site was proposed as a candidate OECM reflects the authors’ overall appraisal following their assessment, rather than a formal decision or definitive classification.

### Knowledge gaps

The review identified several knowledge gaps explicitly reported by the authors of the reviewed studies when assessing potential OECMs. These gaps related to limited evidence on the cost-effectiveness of potential OECMs, uncertainty regarding the representativeness of selected sites, and insufficient empirical data demonstrating that local governance arrangements consistently support positive biodiversity outcomes.

Many studies also highlighted the lack of robust ecological data on site condition, including limited information on species recruitment, dispersal and recolonisation dynamics in disturbed habitats, as well as the environmental drivers influencing these processes. Additional gaps concerned the limited assessment of spatial overlap between potential OECMs and areas of high biodiversity importance, and the scarcity of evaluations quantifying the actual conservation benefits delivered by potential OECMs over time.

Beyond ecological evidence, several studies emphasised gaps in understanding the legal, social and managerial dimensions of OECM identification and recognition, including the need for more participatory and cross-sectoral approaches, improved spatial mapping and connectivity analyses, and clearer consideration of interactions between potential OECMs and broader pressures such as climate change (Table S6, Field #34).

## Discussion

By systematically reviewing and mapping evidence from peer-reviewed and grey literature across realms, sectors and governance regimes globally, this review provides a consolidated overview of how potential OECMs are being identified, interpreted, and evaluated in practice. Given that (a) the OECM concept is still relatively new (CBD 2018) and concerns are raised about the risk of it being insufficiently tapped or integrated into conservation practice (Alves-Pinto et al. 2021; Claudet et al. 2022; Lemieux et al. 2022), and (b) 2030 is fast approaching and the “30 × 30” global biodiversity target is still out of reach (17.55% terrestrial and inland waters, 9.85% marine coverage; UNEP-WCMC and IUCN 2025), synthesizing the available evidence is both timely and necessary to inform future implementation pathways.

This review contributes in two main ways. First, it documents a wealth of information on OECMs, including proposed sites, assessment approaches, and decision-support tools, and consolidates it in the OECMs Database (see Tables S6), which serves as a valuable resource for researchers and policymakers. Secondly, it identifies patterns and recurrent knowledge gaps, offering an empirical basis for strengthening evaluation practices and supporting transparent and credible recognition processes.

This review offers several strengths. First, to our knowledge, it is the only study to date that applies a formal, systematic scoping review methodology to the topic. Previous published studies, including narrative reviews (Cook 2024a, b) and conceptual papers (Himes-Cornell et al. 2022), offer essential insights but do not incorporate the full set of methodological components of a systematic or scoping review, such as predefined research questions, transparent inclusion and exclusion criteria, comprehensive and reproducible search strategies, structured data extraction, and complete reporting of screening outcomes. As a result, they provide valuable thematic or sector-specific perspectives but do not systematically map the empirical evidence base on how potential OECMs are identified and assessed. By applying an established scoping review methodology (Arksey and O’Malley 2005; Levac et al. 2010; Peters et al. 2020a, b) and adhering to PRISMA reporting guidelines (Tricco et al. 2018), this review offers a transparent and replicable synthesis across realms, sectors, and document types. It assembles evidence on the characteristics of potential OECMs, the methods and criteria used to assess them, the sectors involved, and the knowledge gaps identified in the literature, thereby advancing the field beyond conceptual or sector-specific discussions and providing the first systematic global mapping of evidence on potential OECMs.

Second, this review synthesises literature published up to mid-September 2025, capturing a rapidly expanding evidence base, particularly after 2021 (Fig. 2). By including this recent surge in publications, this review provides an up-to-date picture of how the OECM concept is evolving in research and practice.

Third, a further strength is the systematic inclusion of grey literature, which is essential for understanding how OECMs are conceptualised and evaluated in applied settings. Challenges in accessing and reviewing grey literature are well recognised (Mahood et al. 2014; Paez 2017), and this review addressed them through a well-defined search strategy and an a priori review protocol that explicitly set out grey-literature sources and procedures (Petza et al. 2023). This is the first OECM synthesis to comprehensively integrate grey literature, and doing so strengthens the robustness of the findings by reflecting a wider diversity of governance systems, management approaches, and practical experiences.

Several factors contribute to the robustness and reliability of this review: (a) the use of systematic eligibility criteria structured around the PCC—"Participants, Concept, Context" mnemonic (Peters et al. 2020a,b), ensuring consistency in study selection; (b) the use of specialized systematic review software (Covidence 2025) and a team-based screening approach (Levac et al. 2010), reducing bias; (c) the publication of an a priori peer-reviewed protocol (Petza et al. 2023), enhancing transparency and minimizing duplication of research efforts (Page et al. 2021); and (d) the use of advanced data visualization tools to support clearer interpretation of the compiled information (Post et al. 2002; Grainger et al. 2016). The open-access database developed through this review (Table S6) ensures that the compiled knowledge is publicly available, supporting scientific collaboration, evidence-based policymaking, and future research (Molloy 2011; Nosek et al. 2013; Huston et al. 2019). This database compiles the full body of extracted information and, through a structured process, integrates diverse approaches, methodologies, and area-based measures related to potential OECMs. It includes proposed area-based measures, assessment methods and tools, and documented knowledge gaps and policy recommendations on spatial contributions to conservation targets. By assembling these elements in a standardised format, the database provides the first consolidated evidence base on potential OECMs and allows users to explore how studies conceptualise and assess them globally. As an open-access resource, it supports researchers, policy advisors, practitioners, and decision-makers by offering a transparent, unified source to inform scientific analysis, guide policy, and foster collaboration. Ultimately, it serves as a practical tool for advancing understanding of OECMs and their potential role in achieving global spatial conservation targets.

While this review applied a rigorous and transparent methodology, a few limitations should be acknowledged. The language scope of the review was broad, covering major languages of scientific publishing (Di Bitetti and Ferreras 2017; Bahji et al. 2023), i.e., English, French, German, and Spanish, but 19 out of 1624 screened documents were excluded due to linguistic constraints. These comprised mostly Asian languages: 12 studies in Korean, four in Chinese, one in Finnish, one in Indonesian and one in Japanese. Although these studies represent a small proportion of the total, it is possible that some region-specific insights were not captured (Amano et al. 2023). AI translation tools were not used due to concerns about potential biases and inaccuracies in translating complex ecological and policy-related texts (Awadh 2024; Gordon 2024).

Moreover, as is typical for scoping reviews, this study did not critically appraise the methodological quality of included documents (Arksey and O'Malley 2005; Peters et al. 2020a, b). The aim was to map the breadth of available evidence rather than evaluate the rigour of individual studies (Levac et al. 2010).

This review focuses exclusively on potential OECMs documented in the peer-reviewed and grey literature and does not analyse sites already formally recognised and reported to the WD-OECM. This was a deliberate methodological decision, as recognised OECMs would require a separate dataset, search strategy, and analytical framework. As such, the present review should be understood as mapping the available evidence on how potential OECMs are identified and assessed, rather than evaluating the characteristics of sites already recognised as OECMs. Future studies could complement this work by comparing recognised OECMs with those proposed in the literature.

To place the findings in context, the observed patterns should be interpreted in light of existing research and the evolving literature on OECMs. The results of this review demonstrate that although publications on potential OECMs have increased considerably since 2021 (Fig. 2), the evidence base remains sectorally uneven and spatially fragmented, with most studies focusing on early-stage identification rather than robust assessments. Most studies propose or preliminarily examine potential OECMs, while relatively few investigate ecological effectiveness or long-term contributions to biodiversity conservation. This imbalance reflects both the novelty of the OECM concept and the limited availability of standardised methods for evaluating potential OECMs.

Although many studies reported the spatial extent of potential OECMs, the inconsistent reporting of area metrics and lack of standardised spatial data limited comparative analyses across sectors and realms, highlighting the need for more systematic spatial reporting in future OECM assessments. The geographic distribution of the reviewed case studies reveals an uneven global engagement. Countries such as India, China, Colombia, Norway, South Africa, Vietnam, Canada, Mexico, Kenya, and Indonesia dominate the literature, indicating that academic institutions, national agencies, and conservation organisations in these regions have been particularly active in exploring how the OECM concept might be applied in practice. This pattern does not align with the list of countries that have formally recognised and reported OECMs to the WD-OECM, currently including Morocco, Bhutan, Canada, the Cook Islands, Colombia, Algeria, Ecuador, Guernsey, Japan, South Korea, Oman, Peru, the Philippines, Sweden, and Eswatini (UNEP-WCMC and IUCN 2025). The partial overlap suggests that while some countries draw directly on scientific or grey literature assessments when preparing national submissions (as appears to be the case for Canada, Colombia, South Korea, and Japan), others rely primarily on governmental processes, internal assessments, or sector-specific reporting systems not reflected in the literature. Conversely, several countries with extensive research output on OECM assessments, such as India, China, Norway, Vietnam, South Africa, and Mexico, have not yet formally reported OECMs to the WD-OECM, indicating that academic or practitioner-led interest in the concept does not necessarily translate into national recognition and reporting. The divergence between where potential OECMs are most frequently studied and where they are already recognised highlights the need for better coordination and integration among research communities, national reporting processes, and the operational pathways governments use to identify and recognise OECMs. As countries continue to expand their OECM portfolios, strengthening these linkages may help ensure that recognition is both scientifically informed and contextually grounded.

The prominence of potential OECMs with primary conservation objectives in the reviewed literature reflects the types of sites considered most feasible candidates for recognition. Because this review synthesises proposed OECMs, the examples documented are skewed toward areas with conservation objectives, established governance structures, or management systems. These areas are more likely to satisfy OECM criteria and therefore are prioritised in early academic or practitioner assessments. This also explains the strong representation of the environmental sector, which has long-standing conservation mandates, robust management frameworks, and comparatively stronger documentation of ecological outcomes.

By contrast, sites with secondary or ancillary conservation objectives, although central to the conceptual breadth of OECMs, are examined less frequently, often due to limited documentation and monitoring. Within this pattern, our findings reveal that ancillary conservation objectives are most commonly associated with fisheries-related sites. This reflects the fisheries sector early engagement in conceptualising and operationalising OECMs (FAO 2019; Petza et al. 2019; 2023; ICES 2021; Garcia et al. 2021, 2022; FAO 2022; Himnes-Cornell et al. 2022; ICES 2023; Agardy et al. 2025). As a result, fisheries area-based measures with ancillary biodiversity benefits are often better documented and therefore more prominent in early OECM proposals than sites arising from other sectors.

Taken together, these patterns should be interpreted as characteristics of the current evidence base rather than deviations from the intended scope of the OECM concept. They reflect the early stage of OECM exploration, where research has naturally concentrated on sectors with clearer conservation mandates or concrete operational pathways for applying the OECM framework. As empirical work expands and national reporting systems mature, a more diverse array of sites, including those where conservation outcomes arise incidentally or as by-products of other management objectives, is likely to become more prominent in the literature. Strengthening documentation, monitoring, and cross-sectoral engagement will be essential to capture the full diversity of areas that may contribute to global biodiversity targets through OECMs.

Despite the availability of the CBD criteria (CBD 2018), the IUCN guidance (IUCN—WCPA 2019; Jonas et al. 2023) and several sector- and region-specific guidance frameworks (Table S7), methodological practices remain inconsistent and often rely on qualitative approaches, expert judgement, and narrative synthesis of available information. Quantitative methods, including spatial modelling, ecological surveys, remote sensing, and biodiversity metrics, are less common, and most studies do not report indicators of biodiversity condition or change. This heterogeneity limits comparability and hampers consistent evaluation of OECM effectiveness across realms, sectors, and regions.

Patterns of effectiveness reported in the literature vary substantially among sectors and realms. Potential OECMs associated with the environmental, fisheries, and IPLC sectors most frequently emerge as effective in author-reported assessments, likely reflecting the presence of more established governance arrangements, clearer conservation mandates, and comparatively better-documented ecological outcomes in these domains. In contrast, sectors such as forestry, the rural economy, and parts of the public and private sectors show higher uncertainty due to heterogeneity in management practices or insufficient evidence to evaluate outcomes confidently. Differences among realms further underscore the uneven maturity of OECM research: terrestrial studies are often characterised by substantial uncertainty, whereas freshwater and particularly marine studies show a broader mix of effective, uncertain, and mixed outcomes. Across realms and sectors, studies that explicitly applied the CBD criteria tended to report clearer and more positive assessments, indicating that structured evaluation frameworks support more consistent judgments. Although the current evidence does not allow the identification of definitive common attributes of “effective” OECMs, the literature suggests that strong governance, clear conservation objectives, and application of CBD criteria are associated with more favourable evaluations. More empirical, standardised, and cross-sectoral approaches will be essential to strengthen future OECM recognition processes. The association between analysis type and effectiveness category suggests that reported outcomes are, at least partly, shaped by methodological choices. Approaches relying primarily on data synthesis are more frequently associated with uncertainty, reflecting limitations in empirical evidence, whereas spatial and ecological analyses are more often linked to effective or mixed outcomes. This does not imply that certain methods are inherently superior, but rather highlights how methodological depth and data availability influence authors’ confidence in judging sites’ effectiveness. These findings reinforce the need for more consistent and empirically grounded approaches to support clearer and more comparable reporting across studies.Additionally, the review revealed that the use of assessment tools remains limited. Although several decision-support and screening frameworks exist (e.g., CCEA 2018; Shabtay et al. 2019; Jonas et al. 2023), they are applied inconsistently and rarely tested across diverse ecological and governance contexts. This constrains learning and slows progress in operationalising the OECM concept in practice.

Beyond methodological issues, the review highlights important conceptual and practical gaps that hinder the advancement of OECM identification and recognition, as explicitly reported by the authors of the reviewed studies. These include limited understanding of ecological processes, insufficient documentation of governance arrangements and their influence on conservation outcomes, a need for clearer integration of cultural and social values, and inadequate attention to spatial connectivity, representativeness, and the relationship between potential OECMs and high-biodiversity areas.

As more countries work towards formal recognition and reporting OECMs, strengthening consistency, transparency, and empirical grounding in future research will be essential for supporting credible national submissions and ensuring that potential OECMs contribute meaningfully to global biodiversity targets. Collectively, these insights underline the importance of developing more rigorous, harmonised, and multi-dimensional approaches to assessing potential OECMs, highlighting clear priorities for future scientific and policy efforts.

The findings of this scoping review hold significant implications for both policy and research. OECM is a relatively new concept in conservation policy and research, introduced as part of global efforts to improve biodiversity protection and expand conservation beyond traditional protected areas. By elucidating the current state of OECMs and their conservation potential, this review can steer decision-making and help shape the future direction of research and practice in environmental conservation and biodiversity management.

Specifically, for policymakers and conservation managers, this review provides a foundational resource for identifying and recognising potential OECMs, offering insights into their characteristics, governance, and effectiveness. It also provides a comprehensive list of proposed sites and existing assessment tools that can assist practitioners in prioritising locations for further evaluation and supporting national reporting efforts.

Additionally, for researchers, this review highlights the need for straightforward and standardised guidelines and user-friendly, accessible tools that aid in the identification, recognition, and reporting of OECMs. Such tools should be designed to prioritise potential sites and to evaluate their strengths and weaknesses relative to the CBD's OECM criteria. Although the guidance provided by the IUCN (IUCN-WCPA 2019; Jonas et al. 2023; 2024b) is valuable and comprehensive, it requires further framing, quantification, and operationalisation to effectively engage and attract stakeholders from various sectors, including managers and policy decision-makers. Furthermore, these guidelines must be tailored for clarity and applicability to a global audience, ensuring they are widely comprehensible and implementable. Case studies of OECM assessments would be particularly beneficial in illustrating how these guidelines and tools can be applied in practice, providing tangible examples to guide stakeholders and refine processes (Jonas et al. 2024b).

Given that more than half of the case studies assessed reported uncertainty, lack of knowledge, and ineffectiveness, future research should prioritise the development of methodologies and guidelines for standardised assessments of the effectiveness of potential OECMs in achieving in situ conservation outcomes (Gurney et al. 2021; Cook 2024b). This effort could build upon established frameworks used to assess protected area effectiveness, such as the Protected Area Management Effectiveness Framework (PAME; Hockings et al. 2006), developed by the IUCN World Commission on Protected Areas and the various methodologies and tools proposed subsequently based on the PAME framework. These include the Management Effectiveness Tracking Tool (METT) by the World Bank and WWF (Stolton et al. 2007), the Rapid Assessment and Prioritisation of Protected Area Management (RAPPAM) by WWF (Ervin 2003), and the Integrated Management Effectiveness Tool (IMET) by the EU Joint Research Centre and IUCN (Paolini et al. 2016).

This review highlights a significant research gap regarding Criterion D—"Associated ecosystem functions and services and cultural, spiritual, socio-economic and other locally relevant values", underscoring the need to better understand the social dimensions of OECMs. This Criterion emphasises the importance of recognising and safeguarding the deep connections between local communities and their environments, including traditional knowledge, livelihoods, and cultural practices closely tied to ecosystems. Addressing this gap is critical for understanding how OECMs support human well-being, promote social equity, and promote inclusive conservation approaches. Such a focus will help ensure that OECMs not only deliver biodiversity outcomes but also align with the needs and aspirations of the people who depend on and steward these landscapes and seascapes.

## Conclusions

This scoping review provides the first systematic and globally comprehensive synthesis of how proposed OECMs are being identified, assessed, and interpreted across diverse ecological, social, and governance contexts. Our findings highlight a significant rise in engagement with the OECM concept worldwide, but they also reveal persistent asymmetries: research is concentrated in certain countries and sectors, empirical assessment remains limited, and evidence on long-term effectiveness is scarce. The predominance of sites with primary conservation objectives reflects the early stages of OECM exploration, focusing on areas most likely to meet CBD criteria, while the strong representation of fisheries-related ancillary sites highlights the pioneering role of that sector in developing operational pathways for OECM application.

Methodological inconsistencies, including reliance on qualitative assessments, limited use of biodiversity indicators, and low uptake of existing decision-support tools, continue to hinder comparability and weaken evidence-based recognition processes. Conceptual and practical gaps persist, especially regarding governance, social values, and Criterion D considerations, as well as spatial representativeness and ecological connectivity.

Although OECMs have substantial potential to contribute meaningfully to global biodiversity targets, realising this potential requires clearer operational guidance, more consistent and empirically grounded assessment practices, and stronger alignment between research outputs, governance institutions, and national reporting processes.

Looking ahead, several priorities emerge for advancing credible and effective OECM implementation. Policymakers can draw on this review as a consolidated evidence base to guide the identification and recognition of candidate OECMs. Researchers are encouraged to develop more robust, standardised, and operational methodologies for assessing effectiveness, building where appropriate on established protected-areas evaluation frameworks. Strengthened documentation, monitoring, and cross-sectoral engagement will be essential for expanding the diversity of OECMs and ensuring their meaningful and enduring contribution to global biodiversity goals.

## Supplementary Information

Below is the link to the electronic supplementary material.

Supplementary File 1: Figures S1–S10 and Tables S1–S5 and S7 (PDF 1684 KB)

Supplementary File 2: Table S6 (XLSX 340 KB)
