# Supplementary material for: Unlocking the potential of other effective area-based conservation measures (OECMs) for achieving conservation targets: A global scoping review
Source: Ambio. 2026 Feb 7;55(7):1403–25. doi: 10.1007/s13280-025-02341-3 (PMC13230371; doi:10.1007/s13280-025-02341-3)
Supplement: Supplementary file 1 — Figures S1–S10 and Tables S1–S5 and S7 (PDF 1684 KB) [file 13280_2025_2341_MOESM1_ESM.pdf]

Ambio

Supplementary Information

This supplementary information has not been peer-reviewed.

Petza, D., E. Amorim, E. Ben Lamine, F. Colloca, E. Dominguez Crisóstomo, E. Fabbrizzi, S. Fraschetti, I. Fraschetti, I. Galparsoro, S. Giakoumi, M. Kruse, V. Stelzenmüller and S. Katsanevakis. 2026. Unlocking the potential of other effective area-based conservation measures (OECMs) for achieving conservation targets: A global scoping review. *Ambio*. **https://doi.org/10.1007/s13280-025-02341-3**

I. SUPPLEMENTARY FIGURES

A

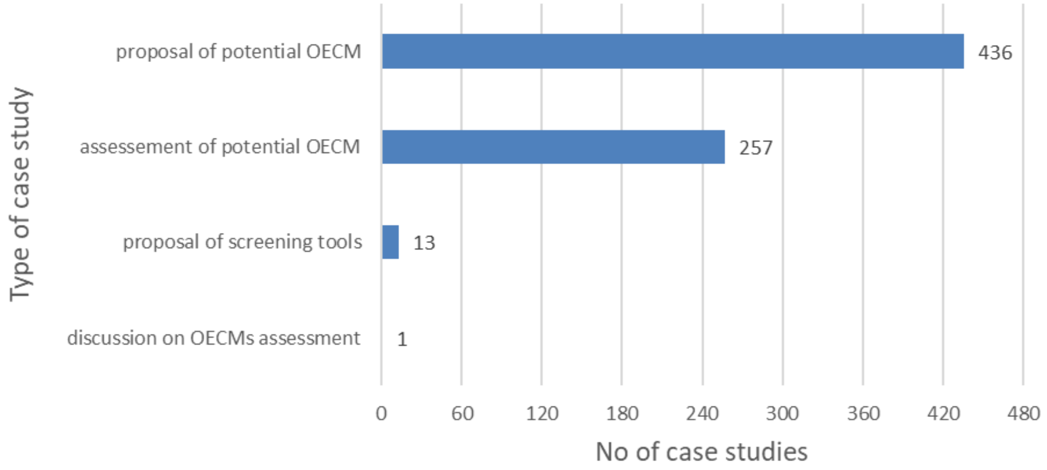

B

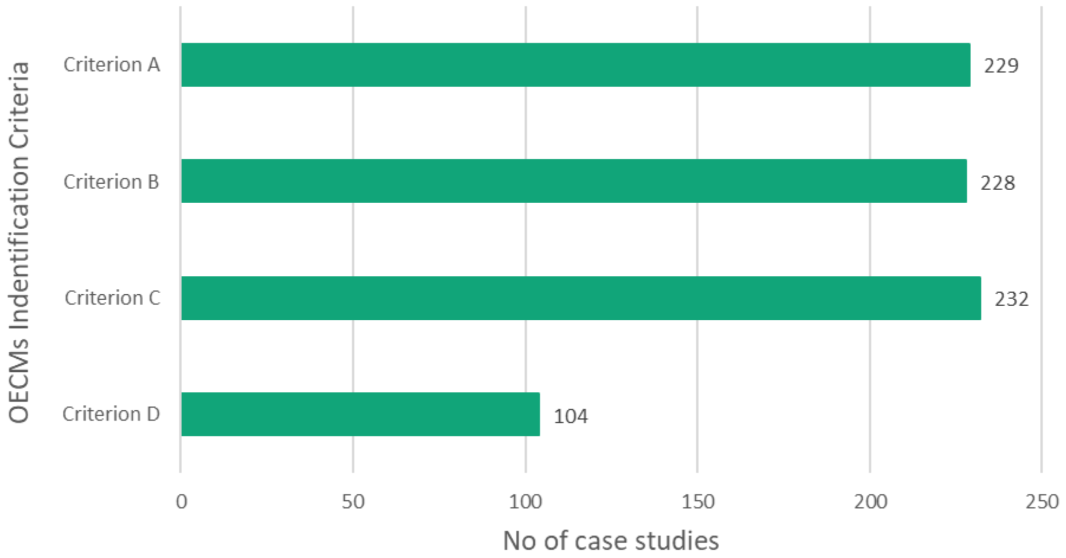

Figure S1. *(A) The number of case studies by type of case study based on its objective and (B) OECMs identification criteria related to the aspects considered or assessed as identified by the CBD Decision 14/8 (CBD, 2018). Criterion A: Area is not currently recognised as a protected area; Criterion B: Area is governed and managed; Criterion C: Achieves sustained and effective contribution to in situ conservation of biodiversity; Criterion D: Associated ecosystem functions and services and cultural, spiritual, socio-economic and other locally relevant values (Fields #36 & 38 of the database; Supplementary Table S6).*

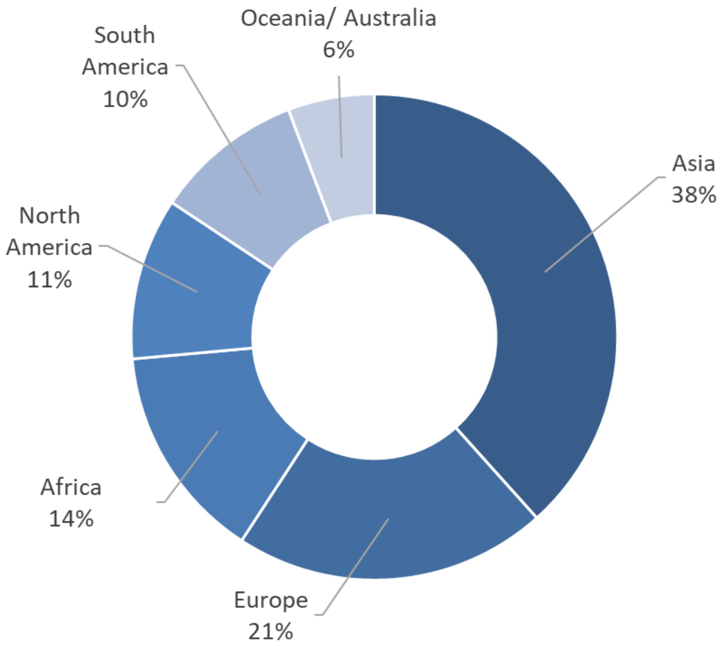

Figure S2. *Proportion of the case studies included in the scoping review by continent (Field #15 of the database; Supplementary Table S6).*

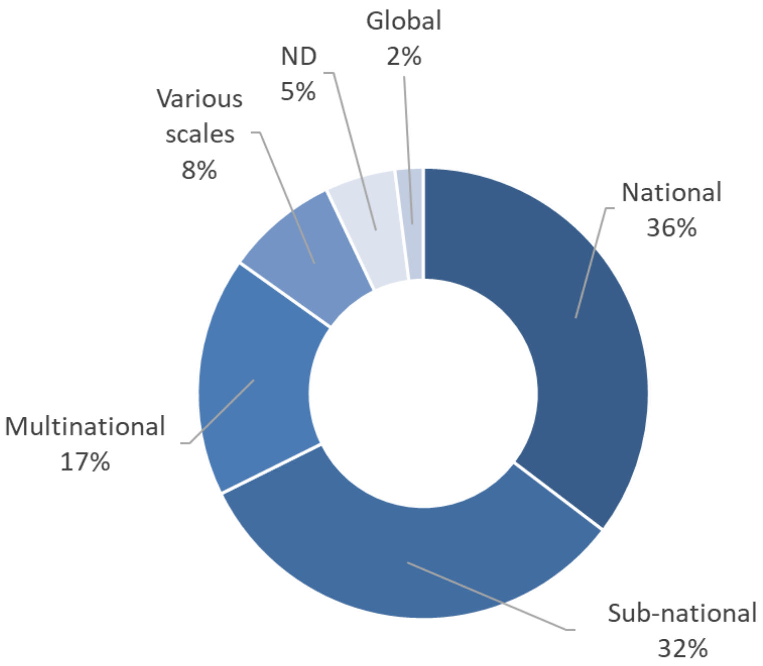

Figure S3. *Proportion of the case studies included in the scoping review by scale. ND = not defined (Field #18 of the database; Supplementary Table S6).*

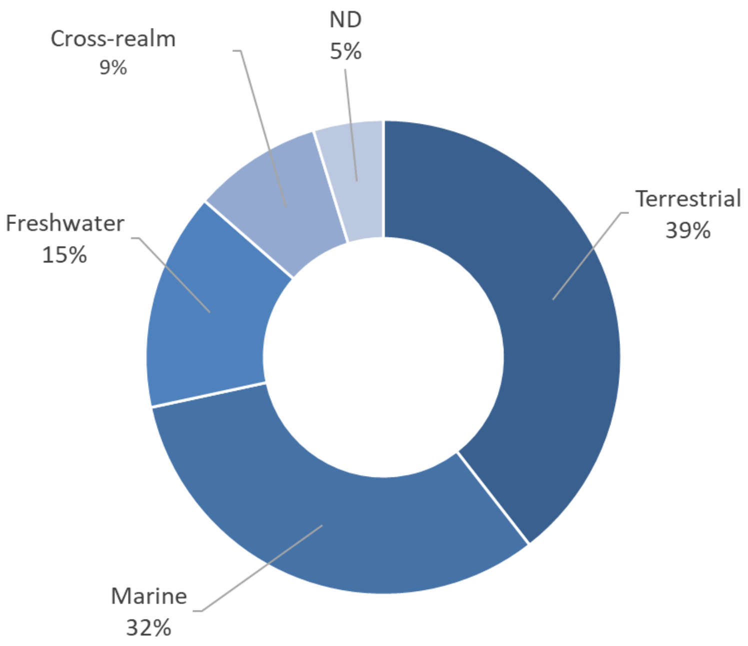

Figure S4. *Proportion (%) of potential OECMs case studies included in the scoping review by realm. ND = not defined (Field #17 of the database; Supplementary Table S6).*

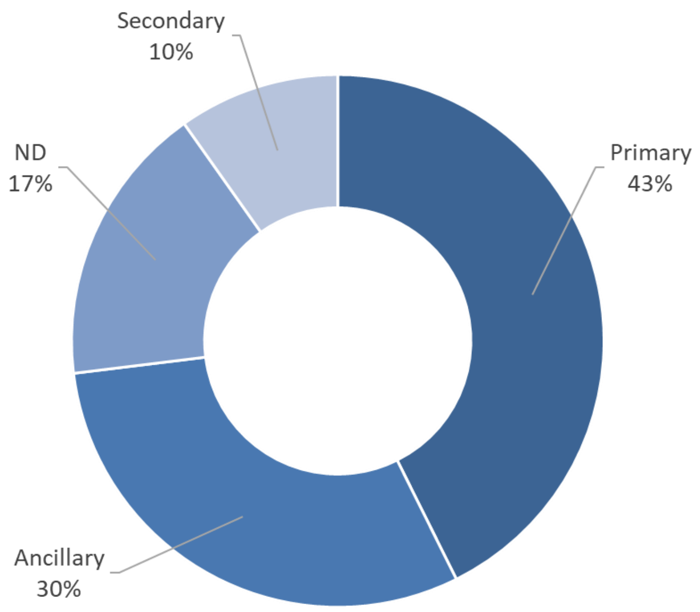

Figure S5. *Proportion of potential OECMs case studies included in the review by conservation objective. ND = not defined (Field #22 of the database; Supplementary Table S6).*

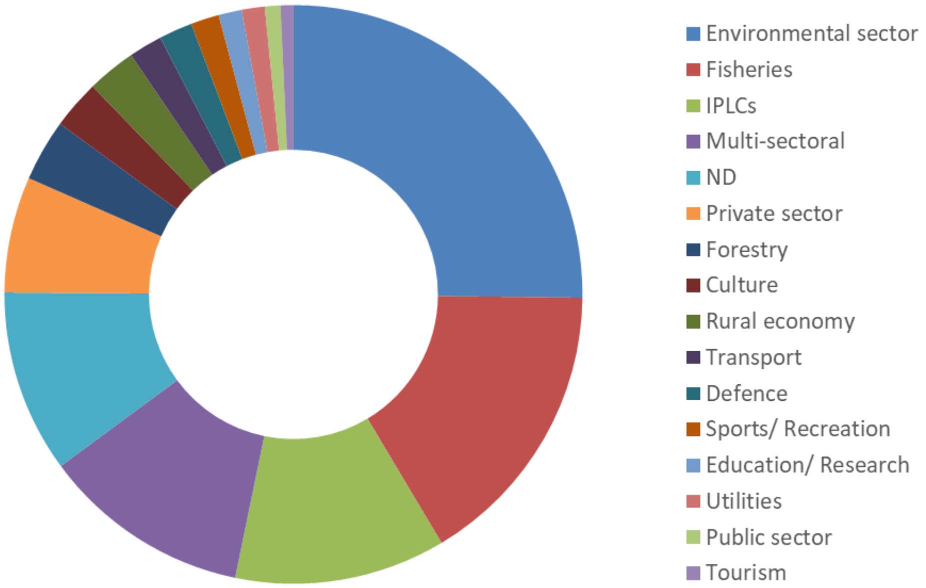

Figure S6. *Proportion of potential OECMs case studies by sector (Field #20 of the database; Supplementary Table S6). The sector classification is based on the International Labour Organisation (https://www.ilo.org/)classification and adequately adjusted to align with the needs of the current study. IPLCs = Indigenous Peoples and Local Communities,Multi-sectoral = involving more than one sector, ND = not defined*

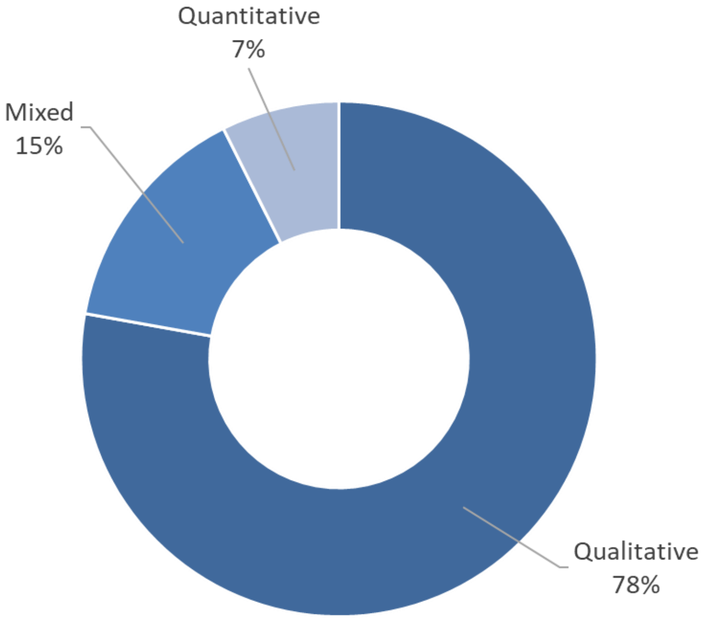

Figure S7. *Proportion of type of* the *research applied for the assessment of potential OECMs (Field #27 of the database; Supplementary Table S6).*

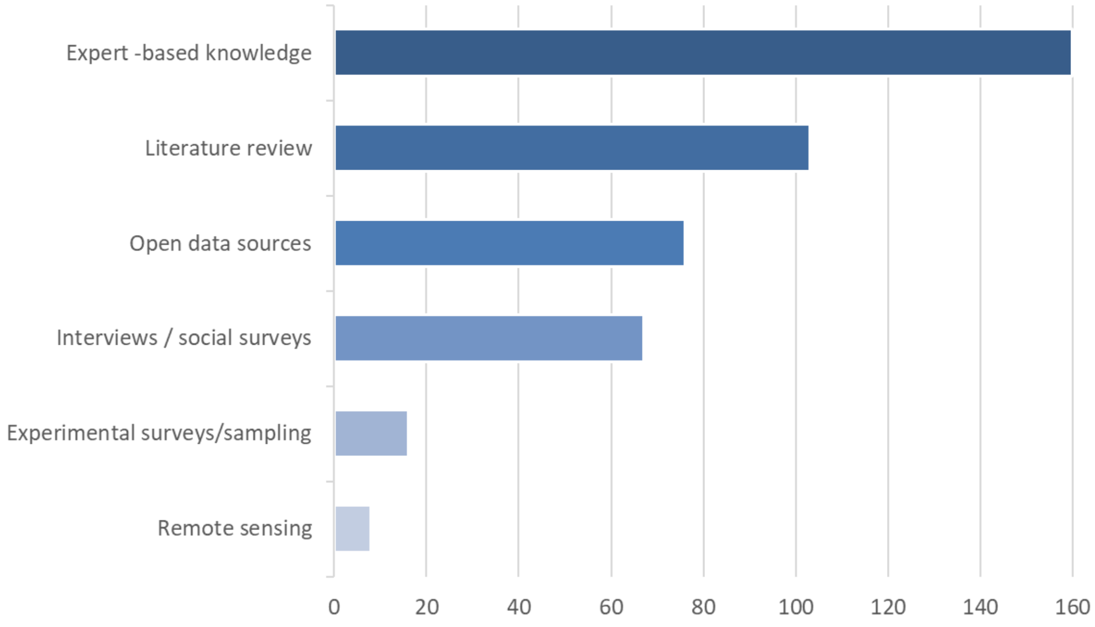

Figure S8. *Number of case studies included in the scoping review by data collection method applied for the potential OECMs assessments (Field #28 of the database; Supplementary Table S6).*

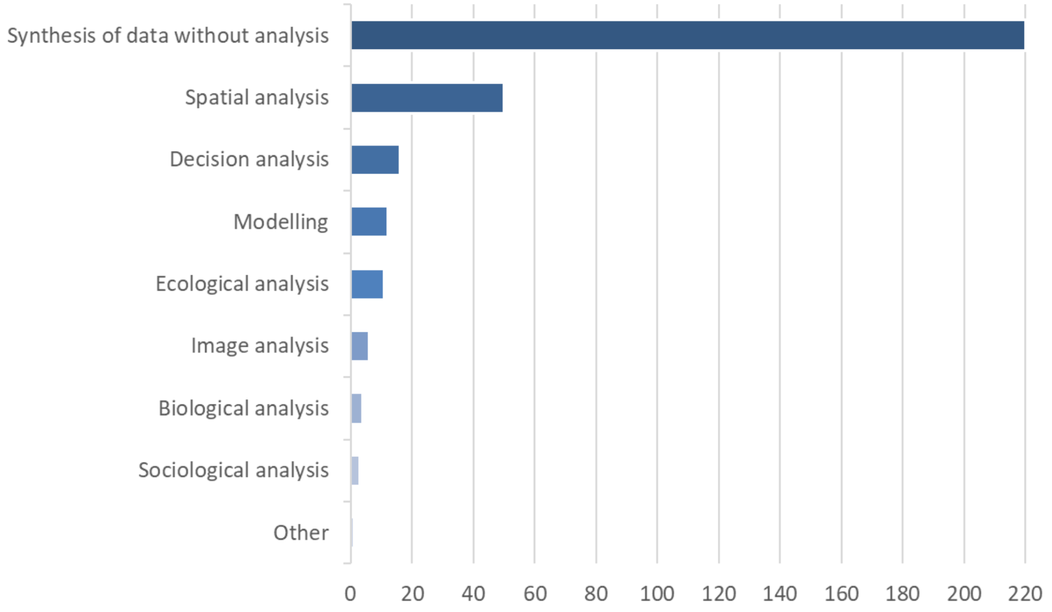

Figure S9. *Number of case studies included in the scoping review by data analysis method applied for the potential OECMs assessments. Other = Inferential statistics (Field #29 of the database; Supplementary Table S6).*

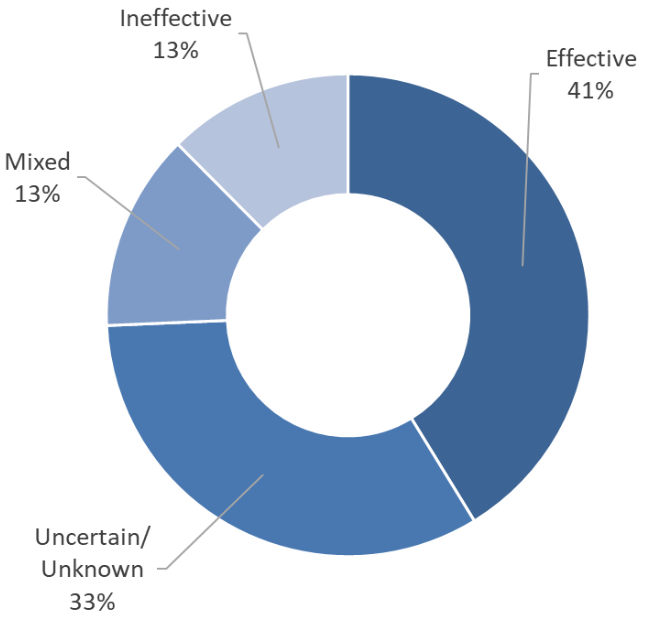

Figure S10. *Proportion of the potential Other Effective area-based Conservation Measures (OECMs) effectiveness reported by the authors of the reviewed studies for the specific aspects or criteria evaluated(Field #32 of the database; Supplementary Table S6).*

II. SUPPLEMENTARY TABLES

Table S1. *Deviations from the published protocol (Petza et al. 2023).*

no	Protocol method (section the protocol)	Deviation from protocol method with justification and type of deviation
**1**	**Aim of the scoping review (§Introduction)**	The aim of the scoping review and its objectives were rephrased to enhance clarity, as suggested by the full-review paper reviewers.<br>*Type of deviation*: modification (clarification)
2	What are the good practices and failures acknowledged in the literature? (§Review question)	As there is no critical appraisal of the studies proposed, it is not possible to assess what is "good practice" or "bad practice". Thus, this research question was omitted from the review.<br>*Type of deviation*: omission
3	(§Review question)	The review question and subquestions of the scoping review were rephrased as follows to enhance clarity, as suggested by the full-review paper reviewers.<br>"The central research question guiding this scoping review was: What is the current knowledge on how potential OECMs are identified, assessed, and reported to contribute to biodiversity conservation targets? To address this, the review explored the following sub-questions: (1) Where are potential OECMs being studied globally? (2) What types of potential OECMs are described (governance, sector, realm, objectives)? (3) What assessment approaches and tools are used? (4) What spatial extent of potential OECMs is documented? (5) What are the reported outcomes (effectiveness, gaps, recommendations)?"<br>*Type of deviation:* modification (clarification)
4	(§Review question)	The following review sub-question was added to align with the review objectives and the data extraction tool fields: "What are the reported outcomes (effectiveness, gaps, recommendations)"<br>*Type of deviation*: addition
5	(§ Inclusion and exclusion criteria and Table 1) Proposing potential OECMs and potential OECMs screening tools were added to the "Concept" of the PCC mnemonic for the formulation of the inclusion criteria.<br>Type of deviation: addition	OECMs, which are referred to by the study as actual OECMs, i.e., which have been identified, recognised and reported at the World Database for Protected Areas (WDPA), were added to the exclusion criteria of the "Participants" of the PCC mnemonic.<br>*Type of deviation:* addition (clarification)
6	(§ Inclusion and exclusion criteria and Table 1)	Proposing potential OECMs and potential OECMs screening tools were added to the "Concept" of the PCC mnemonic for the formulation of the inclusion criteria.<br>*Type of deviation*: clarification (addition)
7	Data extraction from the documents included in the ScR will be carried out using a data extraction tool, i.e., a charting table aligned to the objective and the questions of the ScR (see Extended data (Petza et al., 2023)). (§Methods, Data Extraction).<br>The following categories were provided in the data extraction tool for the field F17 "OECMs sector": transport, offshore energy, fisheries,	For consistency reasons, the classification of the OECMs sector was modified in the final review paper based on the International Labor Organization sectors' classification.<br>The following was used: Commerce; Construction; Education/ Research; Financial services; Forestry; Health services; Hotel, Catering Tourism; Mechanical and electrical engineering; Media, Culture, Graphical; Mining (coal, other mining); Oil and gas production, Oil refining; Public service Fisheries; Maritime (Shipping, Ports, Inland waterways); Textiles, Clothing, Leather and Footwear; Transport (including civil aviation, railways, road transport); Transport

	aquaculture, maritime, tourism, defence, archaeological heritages etc.	equipment manufacturing; Utilities (water, gas, electricity); Rural economy (Agriculture, Plantations and other rural sector, Food, drink and tobacco); Environmental sector; Indigenous Peoples and Local Communities (IPLCs); Defense; Sports and recreation; Renewable energy; Other.<br>*Type of deviation*: modification
8	(§Extended data, Supplementary Table S3 Data extraction tool)	Field #33 “Good practice or failure” was deleted from the data extraction tool following deviation no 1.<br>*Type of deviation*: omission
9	(§Extended data, Supplementary Table S3 Data extraction tool)	The following new fields were added to the data tool to align with the review objectives:<br>#1 “Source” (Scopus, WebOfScience, Scholar Google; Other sources: References search, Stakeholders)<br>#2 “Document code” (free text)<br>#3 “Case study code” (free text)<br>#8 “Abstract” (free text)<br>#36 “Type of case study” (assessment of potential OECMs, proposal of potential OECMs, proposal of screening tool, comparison of screening tools, discussion of potential OECMs assessment)<br>#37 “Specific aspects of potential OECMs considered or assessed” (e.g. biodiversity, management effectiveness, governance etc.)<br>#38 “OECMs criteria related to the aspects considered or assessed” (A: The area is not currently recognised as PAs, B: The area is governed and managed, C: The area achieves sustained and effective contribution to in situ conservation of biodiversity; D: Associated ecosystem functions and services and cultural, spiritual, socio-economic and other locally relevant values are conserved and respected.)<br>*Type of deviation*: addition

Table S2. *Preferred Reporting for Systematic Reviews and Meta-Analyses extension for scoping reviews (PRISMA-ScR) checklist.*

SECTION	ITEM	PRISMA-ScR CHECKLIST ITEM	REPORTED IN SECTION #
**TITLE**			
Title	1	Identify the report as a scoping review.	Title
**ABSTRACT**			
Structured summary	2	Provide a structured summary that includes (as applicable): background, objectives, eligibility criteria, sources of evidence, charting methods, results, and conclusions that relate to the review questions and objectives.	Abstract
**INTRODUCTION**			
Rationale	3	Describe the rationale for the review in the context of what is already known. Explain why the review questions/objectives lend themselves to a scoping review approach.	Introduction
Objectives	4	Provide an explicit statement of the questions and objectives being addressed with reference to their key elements (e.g., population or participants, concepts, and context) or other relevant key elements used to conceptualize the review questions and/or objectives.	Introduction
**METHODS**			
Protocol and registration	5	Indicate whether a review protocol exists; state if and where it can be accessed (e.g., a Web address); and if available, provide registration information, including the registration number.	Materials and methods/ Methodology
Eligibility criteria	6	Specify characteristics of the sources of evidence used as eligibility criteria (e.g., years considered, language, and publication status), and provide a rationale.	Materials and methods/ Inclusion/exclusion criteria & Table 1
Information sources*	7	Describe all information sources in the search (e.g., databases with dates of coverage and contact with authors to identify additional sources), as well as the date the most recent search was executed.	Materials and methods/ Methodology/ Search Strategy
Search	8	Present the full electronic search strategy for at least 1 database, including any limits used, such that it could be repeated.	Materials and methods/ Table 2
Selection of sources of evidence†	9	State the process for selecting sources of evidence (i.e., screening and eligibility) included in the scoping review.	Materials and methods/ Methodology – Study/Source of Evidence Selection
Data charting process‡	10	Describe the methods of charting data from the included sources of evidence (e.g., calibrated forms or forms that have been tested by the team before their use, and whether data charting was done independently or in duplicate) and any processes for obtaining and confirming data from investigators.	Materials and methods/ Methodology – Data Extraction
Data items	11	List and define all variables for which data were sought and any assumptions and simplifications made.	Materials and methods/ Methodology – Data Extraction & Table 3
Critical appraisal of individual sources of evidence§	12	If done, provide a rationale for conducting a critical appraisal of included sources of evidence; describe the methods used and how this information was used in any data synthesis (if appropriate).	Critical appraisal was not performed (not mandatory in Scoping Reviews)
Synthesis of results	13	Describe the methods of handling and summarizing the data that were charted.	Materials and methods/ Methodology – Data Analysis & Presentation
**RESULTS**			

SECTION	ITEM	PRISMA-ScR CHECKLIST ITEM	REPORTED IN SECTION #
Selection of sources of evidence	14	Give numbers of sources of evidence screened, assessed for eligibility, and included in the review, with reasons for exclusions at each stage, ideally using a flow diagram.	Results/ Scoping review workflow and dataset
Characteristics of sources of evidence	15	For each source of evidence, present characteristics for which data were charted and provide the citations.	Results/ Scoping review workflow and dataset
Critical appraisal within sources of evidence	16	If done, present data on critical appraisal of included sources of evidence (see item 12).	Critical appraisal was not performed (not mandatory in Scoping Reviews)
Results of individual sources of evidence	17	For each included source of evidence, present the relevant data that were charted that relate to the review questions and objectives.	Results/ Database
Synthesis of results	18	Summarize and/or present the charting results as they relate to the review questions and objectives.	Results
**DISCUSSION**			
Summary of evidence	19	Summarize the main results (including an overview of concepts, themes, and types of evidence available), link to the review questions and objectives, and consider the relevance to key groups.	Discussion - The role of OECMS as conservation tools
Limitations	20	Discuss the limitations of the scoping review process.	Discussion - The role of OECMS as conservation tools
Conclusions	21	Provide a general interpretation of the results with respect to the review questions and objectives, as well as potential implications and/or next steps.	Discussion - The role of OECMS as conservation tools
**FUNDING**			
Funding	22	Describe sources of funding for the included sources of evidence, as well as sources of funding for the scoping review. Describe the role of the funders of the scoping review.	Funding

JBI = Joanna Briggs Institute; PRISMA-ScR = Preferred Reporting Items for Systematic reviews and Meta-Analyses extension for Scoping Reviews.
* Where *sources of evidence* (see second footnote) are compiled from, such as bibliographic databases, social media platforms, and Web sites.
† A more inclusive/heterogeneous term used to account for the different types of evidence or data sources (e.g., quantitative and/or qualitative research, expert opinion, and policy documents) that may be eligible in a scoping review as opposed to only studies. This is not to be confused with *information sources* (see first footnote).
‡ The frameworks by Arksey and O'Malley (6) and Levac and colleagues (7) and the JBI guidance (4, 5) refer to the process of data extraction in a scoping review as data charting.
§ The process of systematically examining research evidence to assess its validity, results, and relevance before using it to inform a decision. This term is used for items 12 and 19 instead of "risk of bias" (which is more applicable to systematic reviews of interventions) to include and acknowledge the various sources of evidence that may be used in a scoping review (e.g., quantitative and/or qualitative research, expert opinion, and policy document).
From: Tricco et al. (2018)

Table S3. *Details of Scoping Review search strategy per (A) database (name of the database, date of search, search query number of documents returned by the search) and (B) other sources (source category, name of the source, date of search and number of documents returned by search)*

A. Databases

Database 1:	**Scopus**
Date of search:	September 3, 2025
Query:	TITLE-ABS-KEY ("other effective area-based conservation measure*" OR "other effective area based conservation measure*" OR "other conservation measure*" OR "OECM*" OR "OEABCM*")
Results:	564 documents
Database 2:	**Web of Science – Core Collection**
Date of search:	September 3, 2025
Query:	TS=("other effective area-based conservation measure*" OR "other effective area based conservation measure*" OR "other conservation measure*" OR "OECM*" OR "OEABCM*")
Results 1:	359 documents
Database 3	**Scholar Google**
Date of search:	September 3, 2025
Query	conservation ("other effective area-based conservation measure*" OR "other effective area based conservation measure*" OR "other conservation measure*" OR "OECM*" OR "OEABCM*")
Results:	2136 documents (only the first 200 hits were considered)

B. Other sources

**Source Category**	**Source**	**Date**	**Articles retrieved**
Organisational Libraries	IUCN Library	20/9/2025	21
	FAO Knowledge repository	20/9/2025	243
	ICES Library	20/9/2025	8
	UNEP Knowledge repository	23/9/2025	5
Organisational Websites	Protected Planet / WDPA - Resources	20/9/2025	0
	WWF infrastructure and nature resource library	23/9/2025	0
	Conservation of Arctic Flora and Fauna, CAFF	28/9/2025	0
	IUCN WCPA OECMs Specialist Group Website	23/9/2025	16
Preprint Archive	bioRxiv	20/9/2025	58
	OSF Preprints	23/9/2025	2
	EcoEvoRxiv	23/9/2025	87
	EarthArXiv	23/9/2025	7
	AgriRxiv	23/9/2025	106
Thesis/ Dissertation Archives	Proquest	20/9/2025	100
	Open Access Theses and Dissertations - OATD	20/9/2025	6
	National Archiver of PhD Theses	23/9/2025	1
	The Networked Digital Library of Theses and Dissertations (NDLTD)	28/9/2025	0
	EThOS	28/9/2025	94
	Theses Canada	28/9/2025	1
	WorldCat	28/9/2025	100
Document/ Data repositories	Zenodo	28/9/2025	32
Web-based Engines	Google	28/9/2025	100
Citation Searching	-	-	17
Stakeholder's Suggestions	-	-	3

Table S4. *Data extraction tool.*

Field	Title	Answer	Question	Remarks/ guidance to reviewers (format, definitions, examples, etc.)
F1	Source	Scopus, WebOfScience, Scholar Google; Other sources - References search, Stakeholders/ Experts, FAO knowledge repository, Google, ICES library, IUCN library, WorldCat	*What is the source of the document?*	-
F2	Document code	#01-#99	*What is the code of the document?*	A code attributed by the authors to each document included in the ScR.
F3	Case study code	CS001-CS694	*What is the code of the case study?*	A code attributed by the authors to each case study included in the ScR.
F4	Author(s)	free text	*Who are the authors of the document?*	Last name, first name
F5	Title	free text	*What is the title of the document?*	Full title
F6	Year of publication	free text	*What is the year of publication of the document?*	YYYY (4 digits) e.g., 2021
F7	Journal	free text	*What is the title of the journal?*	Full title (not abbreviated)
F8	Abstract	Free text	*What is the abstract of the document (if any)?*	-
F9	Keywords	free text	*Which are the keywords of the document?*	-
F10	DOI	free text	*What is the DOI of the document (if available)?*	Add DOI in the cases of peer-reviewed articles, e.g., 10.11124/JBISRIR-D-19-00434
F11	URL	free text	*What is the URL of the document (if available)?*	Add URL in the cases of grey literature retrieved via the internet (mandatory when DOI is not available).
F12	Literature category	peer-reviewed literature; grey literature	*Which is the literature category of the document?*	-
F13	Literature type	article; booklet; compilation of case studies; Conference Book of Abstracts meeting summary; preprint;	*Which is the literature type of document?*	-

		organizational paper; report; short communication; technical paper/ report; thesis		
F14	Language	English; Spanish; German; Italian; Greek; French	*What is the language of the document?*	-
F15	Continent	Africa; Antarctica; Asia; Europe; North America; Oceania/Australia; South America; more than one continent; global	*Which is the continent where the study takes place?*	-
F16	Country	Afghanistan, Albania, Algeria, Andorra, Angola, Antigua and Barbuda, Argentina, Armenia, Australia, Austria, Azerbaijan, Bahamas, Bahrain, Bangladesh, …	*Which is the country where the study is located?*	-
F17	Realm	terrestrial; freshwater; marine	*Which is the realm of the potential OECM?*	-
F18	Scale	Global, Multi-national, National, Sub-national	*Which is the scale where the study takes place?*	
F19	OECM designation name	free text	*What is the name of the potential OECM?*	Provide the name as it is reported by the study.
F20	OECM sector	Commerce; Construction; Education/ Research; Financial services; Forestry; Health services; Hotel, Catering Tourism; Mechanical and electrical engineering; Media, Culture, Graphical; Mining (coal, other mining); Oil and gas production, Oil refining; Public service<br>Fisheries; Maritime (Shipping, Ports, Inland waterways); Textiles, Clothing, Leather and Footwear; Transport (including civil aviation, railways, road transport); Transport equipment manufacturing; Utilities (water, gas, electricity); Rural economy (Agriculture, Plantations and other rural sector, Food, drink and tobacco); Environmental sector; Indigenous Peoples and Local Communities (IPLCs); Defense; Sports and recreation; Renewable energy; Other.	*Which sector established the potential OECM?*	-

F21	Rationale	free text	*What is the rationale for the designation of the potential OECM?*	Mention (copy-paste from the manuscript) the specific rationale for the designation of the OECM.
F22	Type of conservation objective	primary; secondary; ancillary	*Which is the type of conservation objective of the potential OECM?*	Primary conservation: refers to areas that may meet all elements of the IUCN definition of a protected area, but which are not officially designated as such because the governance authority does not want the area to be recognized or reported as a protected area.<br>Secondary conservation: is achieved through the active management of an area where biodiversity outcomes are a secondary management objective.<br>Ancillary conservation: refers to areas that deliver in-situ conservation as a by-product of management activities, even though biodiversity conservation is not a management objective (IUCN-WCPA, 2019)
F23	Potential OECM area	free text (number)	*What is the area of the potential OECM (in km^2)?*	Provide the area (in km^2) of the OECM
F24	Potential OECM number	free text (number)	*How many potential OECMs were tested by the study?*	Provide the number of potential OECMs tested.
F25	Methodology	free text	*What is the methodology followed for the assessment of the potential OECMs?*	Briefly describe the methodology.
F26	CBD OECMs criteria	Yes; No	*Did the study test the potential OECM against the CBD OECM criteria?*	The CBD OECMs criteria are foreseen by CBD Decision 14/8
F27	Type of research	quantitative; qualitative; mixed	*What is the type of research applied?*	-
F28	Data collection method	experimental surveys/sampling; expert-based knowledge; fishers' local ecological knowledge; interviews/ social surveys; literature review; open data sources; remote sensing	*Which is the method for the data collection applied?*	-

F29	Data analysis method	fisheries analysis; ecological analysis; modeling; biological analysis; spatial analysis; sociological analysis; economic analysis; physiological or behavioral analysis; physicochemical analysis; image analysis; genetic analysis; decision analysis; biochemical analysis; synthesis of data without analysis	*Which is the method for the data analysis applied?*	-
F30	Metrics	free text	*What are the metrics used for the assessment of potential OECMs (if any)?*	-
F31	Key findings	free text	*What are the key findings of the study?*	Briefly describe the key findings of the study.
F32	OECMs effectiveness	effective; ineffective; neutral; mixed; uncertain; unknown	*According to the study's authors, was the measure deemed effective for the aspects or criteria assessed?*	-
F33	Candidate OECM	Yes; No; Maybe; Not known/ metnioned	*Is the potential OECM proposed as a candidate OECM by the study's authors?*	-
F34	Gaps of knowledge	free text	*What are the gaps of knowledge identified by the study (if any)?*	Briefly describe the gaps of knowledge identified by the study.
F35	Policy recommendations	true; false	*What are the policy recommendations proposed by the study (if any)?*	Briefly describe the policy recommendations proposed by the study.
F36	Type of case study	assessment of potential OECMs; proposal of potential OECMs; proposal of screening tool; comparison of screening tools; discussion of potential OECMs assessment	*What is the type of study?*	-
F37	Specific aspects of the measures considered or assessed	Free text	*What are the specific aspects of the measures that were considered or assessed?*	-

F38	OECM criteria related to the aspects considered or assessed	A, B, C, D	*Which OECM identification criteria were considered by the case study?*	A: The area is not currently recognised as PAs, B: The area is governed and managed, C: The area achieves sustained and effective contribution to in situ conservation of biodiversity; C: Associated ecosystem functions and services and cultural, spiritual, socio-economic and other locally relevant values are conserved and respected.

Table S5. *List of excluded documents during the full-text review stage (authors, title, published year, journal, volume, issue, pages, DOI, abstract, reason for exclusion).*

No	Authors	Title	Published Year	Journal	Volume	Issue	Pages	DOI	Reason for exclusion
1	Pusparini, W.; Cahyana, A.; Grantham, H.S. et al.	A bolder conservation future for Indonesia by prioritising biodiversity, carbon and unique ecosystems in Sulawesi	2023	*Scientific Reports*	13	1	-	10.1038/s41598-022-21536-2	Documents that do not assess or propose potential OECMs
2	Peng, S.; Hu, R.; Velazco, S.J.E et al.	Preserving the woody plant tree of life in China under future climate and land-cover changes	2022	*Proceedings of the Royal Society B: Biological Sciences*	289	1988	-	10.1098/rspb.2022.1497	Documents that do not assess or propose potential OECMs
3	Kumagai, J.A.; Favoretto, F.; Pruckner, S.; et al.	Habitat Protection Indexes - new monitoring measures for the conservation of coastal and marine habitats	2022	*Scientific Data*	9	1	-	10.1038/s41597-022-01296-4	Documents that do not assess or propose potential OECMs
4	Lemieux, C.J.; Kraus, D.T.; Beazley, K.F.	Running to stand still: The application of substandard OECMs in national and provincial policy in Canada	2022	*Biological Conservation*	275	-	-	10.1016/j.biocon.2022.109780	Documents that do not assess or propose potential OECMs
5	Garcia, S.M.; Rice, J.; Himes-Cornell, A.; et al.	OECMs in marine capture fisheries: Key implementation issues of governance, management, and biodiversity	2022	*Frontiers in Marine Science*	9	-	-	10.3389/fmars.2022.920051	Documents that do not assess or propose potential OECMs
6	Claudet, J.; Ban, N.C.; Blythe, J. et al.	Avoiding the misuse of other effective area-based conservation measures in the wake of the blue economy	2022	*One Earth*	5	9	969-974	10.1016/j.oneear.2022.08.010	Documents that do not assess or propose potential OECMs
7	Himes-Cornell, A.; Lechuga SÃ¡nchez, J.F.; Potter, C. et al.	Reaching Global Marine Biodiversity Conservation Goals With Area-Based Fisheries Management: A Typology-Based Evaluation	2022	*Frontiers in Marine Science*	9	-	-	10.3389/fmars.2022.932283	Documents that do not provide sufficient details on potential OECMs

8	Pascher, K.; Å vara, V.; Jungmeier, M.	Environmental DNA-Based Methods in Biodiversity Monitoring of Protected Areas: Application Range, Limitations, and Needs	2022	*Diversity*	14	6	-	10.3390/d14060463	Documents that do not assess or propose potential OECMs
9	Wu, L.; Wang, Y.; Mo, X. et al.	Shifted to the South, Shifted to the North, but no Expansion: Potential Suitable Habitat Distribution Shift and Conservation Gap of the Critically Endangered Baerâ€™s Pochard (Aythya baeri)	2022	*Remote Sensing*	14	9	-	10.3390/rs14092171	Documents that do not assess or propose potential OECMs
10	Ma, J.; Tam, C.; Li, T. et al.	Sacred natural sites classification framework based on ecosystem services and implications for conservation	2022	*Conservation Science and Practice*	4	4	-	10.1111/csp2.12638	Documents that do not assess or propose potential OECMs
11	Dudley, N.; Kettunen, M.; Gorricho, J. et al.	Area-based conservation and the Sustainable Development Goals: a review	2022	*Biodiversity*	23	45355	146-151	10.1080/14888386.2022.2150313	Documents that do not assess or propose potential OECMs
12	Zannini, P.; Frascaroli, F.; Nascimbene, J. et al.	Sacred natural sites and biodiversity conservation: a systematic review	2021	*Biodiversity and Conservation*	30	13	3747-3762	10.1007/s10531-021-02296-3	Documents that do not assess or propose potential OECMs
13	Geldmann, J.; Deguignet, M.; Balmford, A. et al.	Essential indicators for measuring site-based conservation effectiveness in the post-2020 global biodiversity framework	2021	*Conservation Letters*	14	4	-	10.1111/conl.12792	Documents that do not assess or propose potential OECMs
14	Quintana, A.C.E.; Giron-Nava, A.; Urmy, S.et al.	Positive Social-Ecological Feedbacks in Community-Based Conservation	2021	*Frontiers in Marine Science*	8	-	-	10.3389/fmars.2021.652318	Documents that do not assess or propose potential OECMs
15	Alves-Pinto, H.; Geldmann, J.; Jonas, H. et al.	Opportunities and challenges of other effective area-based conservation measures (OECMs) for biodiversity conservation	2021	*Perspectives in Ecology and Conservation*	19	2	115-120	10.1016/j.pecon.2021.01.004	Documents that do not assess or propose potential OECMs

16	Lemieux, C.J.; Gray, P.A.	How Canada â€œhamburger manufacturedâ€ its way to marine protected area success and a more effective and equitable way forward for the post-2020 conservation agenda	2020	*Journal of Environmental Studies and Sciences*	10	4	483-491	10.1007/s13412-020-00627-4	Documents that do not assess or propose potential OECMs
17	Teff-Seker, Y.; Mackelworth, P.C.; Vega FernÃ¡ndez, T. et al.	Do Alternative Dispute Resolution (ADR) and Track Two Processes Support Transboundary Marine Conservation? Lessons From Six Case Studies of Maritime Disputes	2020	*Frontiers in Marine Science*	7	-	-	10.3389/fmars.2020.593265	Documents that do not assess or propose potential OECMs
18	Trew, B.T.; Grantham, H.S.; Barrientos, C. et al.	Using Cumulative Impact Mapping to Prioritize Marine Conservation Efforts in Equatorial Guinea	2019	*Frontiers in Marine Science*	6	-	-	10.3389/fmars.2019.00717	Documents that do not assess or propose potential OECMs
19	Johnson, D.E.; Rees, S.E.; Diz, D. et al.	Securing effective and equitable coverage of marine protected areas: The UK's progress towards achieving Convention on Biological Diversity commitments and lessons learned for the way forward	2019	*Aquatic Conservation: Marine and Freshwater Ecosystems*	29	S2	181-194	10.1002/aqc.3065	Documents that do not assess or propose potential OECMs
20	Lemieux, C.J.; Gray, P.A.; Devillers, R. et al.	How the race to achieve Aichi Target 11 could jeopardize the effective conservation of biodiversity in Canada and beyond	2019	*Marine Policy*	99	-	312-323	10.1016/j.marpol.2018.10.029	Documents that do not assess or propose potential OECMs
21	Mitchell, B.A.; Fitzsimons, J.A.; Stevens, C.M.; Wright, D.R.	PPA or OECM? Differentiating between privately protected areas and other effective area-based conservation measures on private land	2018	*Parks*	24	Special issue	49-60	10.2305/IUCN.CH.2018.PARKS-24-SIBAM.en	Documents that do not assess or propose potential OECMs
22	Jonas, H.D.; Lee, E.; Jonas, H.C. et al.	Will'other effe ctive area-based conservation measures' increase recognition and support for ICCAs?	2017	*Parks*	23	2	63-78	10.2305/iucn.ch.2017.parks-23-2hdj.en	Documents that do not assess or propose potential OECMs

23	Laffoley, D.; Dudley, N.; Jonas, H. et al.	An introduction to â€˜other effective area-based conservation measuresâ€™ under Aichi Target 11 of the Convention on Biological Diversity: Origin, interpretation and emerging ocean issues	2017	*Aquatic Conservation: Marine and Freshwater Ecosystems*	27	-	130-137	10.1002/aqc.2783	Documents that do not assess or propose potential OECMs
24	Juffe-Bignoli, D.; Harrison, I.; Butchart, S.H.M. et al.	Achieving Aichi Biodiversity Target 11 to improve the performance of protected areas and conserve freshwater biodiversity	2016	*Aquatic Conservation: Marine and Freshwater Ecosystems*	26	-	133-151	10.1002/aqc.2638	Documents that do not assess or propose potential OECMs
25	RodrÃguez-RodrÃguez, D.; RodrÃguez, J.; Abdul Malak, D. et al.	Marine protected areas and fisheries restricted areas in the Mediterranean: Assessing "actual" marine biodiversity protection coverage at multiple scales	2016	*Marine Policy*	64	-	24-30	10.1016/j.marpol.2015.11.006	Documents that do not assess or propose potential OECMs
26	Sykes, Rachel E; K O, Helen M; Juffe-bignoli, Diego; Metcalfe et al.	Developing a framework to improve global estimates of conservation area coverage	2022	-	-	-	-	10.32942/OSF.IO/SXMK5	Documents that do not assess or propose potential OECMs
27	LARREA-ALCAZAR, D M; et al	Uneven representation of biogeographical regions in Bolivia's protected areas and indigenous territories.	2016	*EcologÃa en Bolivia*	51	2	141-156	-	Documents that do not assess or propose potential OECMs
28	ICES	Working Group on Marine Benthal and Renewable Energy Developments (WGMBRED)	2021	*ICES Scientific Reports.*	3	63	-	-	Documents that do not assess or propose potential OECMs
29	Lalonde, Suzanne; Abashidze, Aslan; Solntsev, Alexander	Marine Protected Areas and Other Effective Area-based Conservation Measures	2022	*Arctic Review on Law and Politics*	13	0	312â€“337-312â€“337	10.23865/ARCTIC.V13.3352	Documents that do not assess or propose potential OECMs

30	Farquhar, Samantha; Allison, Eddie; Himes-Cornell, Amber; Anderson, Chris; Ota, Yoshi	Implications of â€˜other effective area-based conservation measuresâ€™ for marine conservation	2019	-	-	-	-	-	Documents that do not provide sufficient details on potential OECMs
31	Carroll, Carlos; Ray, Justina C.	Maximizing the effectiveness of national commitments to protected area expansion for conserving biodiversity and ecosystem carbon under climate change	2021	*Global Change Biology*	27	15	3395-3414	10.1111/GCB.15645	Documents that do not assess or propose potential OECMs
32	Hilty, Jodi; Worboys, Graeme L.; Keeley, Annika et al.	Guidelines for conserving connectivity through ecological networks and corridors	2020	-	-	-	-	10.2305/IUCN.CH.2020.PAG.30.en	Documents that do not assess or propose potential OECMs
33	Sellheim, Nikolas	The Growth of Local Recognition in Biodiversity Protection Through Other Effective Area-based Conservation Measures	2019	*Revista Tribuna Internacional*	8	15	-	10.5354/0719-482X.2019.52376	Documents that do not assess or propose potential OECMs
34	UNU-IAS and IGES	Sustainable Use of Biodiversity in Socio-ecological Production Landscapes and Seascapes and its Contribution to Effective Area-based Conservation (Satoyama Initiative Thematic Review vol. 4)	2018	-	-	-	-	-	Documents that do not assess or propose potential OECMs
35	Sellheim, N	TheGrowth of Local Recognition in Biodiversity Protection through OtherEffectiveArea-BasedConservationMeasures.ASpecialEmphasisontheArctic	2019	*Revista Tribuna Internaciona*	8	15	-	-	Documents that do not assess or propose potential OECMs

36	Sparling, Abigail	An opportunity for policy change? Creating new space for conservation through marine 'other effective area-based conservation measures' in Canada	2020	-	-	-	-	-	Documents that do not assess or propose potential OECMs
37	Farquhar, Samantha D; Santos, Maria J.	Maritime Archaeology and Marine Conservation: The need for synergy in an uncertain future	2019	-	-	-	-	-	Documents that do not assess or propose potential OECMs
38	Aquerreta, R.; Vivien, L.	Harnessing the socio-ecological potential of mountain biosphere reserves for biodiversity conservation: policy brief	2022	-	-	-	-	-	Documents that do not assess or propose potential OECMs
39	Gurney, G.G.; Darling, E.S.; Ahmadia, G.N.; Agostini, V.N. et al.	Biodiversity needs every tool in the box: use OECMs	2021	*Nature*	595	7869	646-649	10.1038/d41586-021-02041-4	Documents that do not assess or propose potential OECMs
40	Moraes, O.	Blue carbon in area-based coastal and marine management schemesâ€“a review	2019	*Journal of the Indian Ocean Region*	15	2	193-212	10.1080/19480881.2019.1608672	Documents not retrieved
41	W.M.M., Bandara; I.D.C., Jayasinghe; S.S.T., Samarasinghe et al	Examination of Effectiveness of Environmentally Sensitive Areas in Conservation of Biodiversity outside Protected Areas in Sri Lanka	2022	*Proceedings of International Forestry and Environment Symposium*	26	-	-	10.31357/FESYMPO.V26.5679	Documents not retrieved
42	Clara Matallana; Alexandra Areiza; Clara Solano et al.	Analysis of governace types and the role of communities on other effective area-based conservation measures (OECM) in Colombia	-	-	-	-	-	-	Documents that do not provide sufficient details on potential OECMs
43	Arenas-Castro, Alexandra; Matallana-TobÃ³n, Clara L.; Solano, Clara et al.	Other effective area-based conservation measures (OECM): The case of Colombia	-	-	-	-	-	-	Documents that do not provide sufficient details on potential OECMs

44	Caballero-Rico, F.C.; Roque-HernÃ¡ndez, R.V.; de la Garza Cano, R.; Arvizu-SÃ¡nchez, E.	Challenges for the Integrated Management of Priority Areas for Conservation in Tamaulipas, MÃ©xico	2022	*Sustainability (Switzerland)*	14	1	-	10.3390/su14010494	Documents that do not assess or propose potential OECMs
45	Dudley, N.; Stolton, S.	Leaving space for nature: The critical role of area-based conservation	2020	*Leaving Space for Nature: The Critical Role of Area-Based Conservation*	-	-	1-193	10.4324/9780367815424	Documents not retrieved
46	Kharumnuid, W.; Bharucha, E.	Management practices for biodiversity conservation through community conserved areas in Meghalaya, India	2020	*Indian Journal of Ecology*	47	1	226-234	-	Documents not retrieved
47	Lalonde, S.	Marine protected area networks at the Poles	2020	*Research Handbook on Polar Law*	-	-	346-370	10.4337/9781788119597.00024	Documents not retrieved
48	Spiliopoulou, Konstantina	Tracking changes in protection of greek key biodiversity areas	2022	-	-	-	-	10.12681/eadd/52994	Documents not retrieved
49	Dunbar, William; Subramanian, Suneetha M.; Yanagiya, Makiko	Recognising and Supporting the Role of Culture in Effective Area-based Conservation	2022	-	-	-	-	10.53326/NRLK9587	Documents not retrieved
50	Wang, L.; Lu, X.-Q.; Liu, L. et al.	Opportunities, challenges and policy analysis of other effective area-based conservation measures (OECMs) for biodiversity conservation	2021	*Journal of Ecology and Rural Environment*	37	9	1122-1128	10.19741/j.issn.1673-4831.2021.0152	Documents published in languages other than the reviewers' language competency
51	Heinonen, M; Alanen, A	Areas supporting the protected area network and safeguarding biodiversity in FinlandOECM Working Group proposal	2022	*Finnish Ministry of the Environment*	-	-	148-148	-	Documents published in languages other than the reviewers' language competency

52	Jonas, HD; Bingham, HC; Bennett, NJ et al.	Global status and emerging contribution of other effective area-based conservation measures (OECMs) towards the '30x30' biodiversity Target 3	2024	*Frontiers in Conservation Science*	5	-	1447434	10.3389/fcosc.2024.1447434	Documents that do not assess or propose potential OECMs
53	Arredondo, CA; Fabra, YG; Jaramillo, ACO	Challenges in the implementation of other Conservation (OMEC) in Colombia OECMs	2023	*Justicia*	28	43	125-136	10.17081/just.28.43.5726	Documents that do not assess or propose potential OECMs
54	Lewis, AH; Gottlieb, B; Wilson, B. et al.	Coverage and beyond: how can private governance support key elements of the Global Biodiversity Framework's Target 3?	2023	*Frontiers in Conservation Science*	4	-	1303801	10.3389/fcosc.2023.1303801	Documents that do not assess or propose potential OECMs
55	Pulido, KGR; Velazco, SJE	On protected areas and other effective area-based conservation measures to conserve biodiversity. Exploring their contribution to Colombian snakes	2025	*Perspectives in Ecology and Conservation*	23	-	110–120	10.1016/j.pecon.2025.04.002	Documents that do not assess or propose potential OECMs
56	Sengupta, A; Bhan, M; Bhatia, S. et al.	Realizing 30 x 30 in India: The potential, the challenges, and the way forward	2024	*Conservation Letters*	17	-	e13004	10.1111/conl.13004	Documents that do not assess or propose potential OECMs
57	Pearson, N; Thompson, BS	Saving two fish with one wreck: Maximizing synergies in marine biodiversity conservation and underwater cultural heritage protection	2023	*Marine Policy*	-	152	105613	10.1016/j.marpol.2023.105613	Documents that do not assess or propose potential OECMs
58	Chung, HSE	Implications and Lessons Learnt from Taiwan Marine Biodiversity Governance Transformation	2024	*Coastal Management*	52	6	431–448	10.1080/08920753.2025.2443988	Documents that do not assess or propose potential OECMs
59	Bryndum-Buchholz, A; Eddy, TD; Fisher, JAD	Assessing indirect biodiversity conservation benefits of fisheries closures in the Gulf of St. Lawrence, Canada	2025	*PLOS ONE*	20	1	e0316754	10.1371/journal.pone.0316754	Documents that do not assess or propose potential OECMs

60	Mouillot, D; Velez, L; Albouy, C et al.	The socioeconomic and environmental niche of protected areas reveals global conservation gaps and opportunities	2024	*Nature Communications*	15	-	9007	10.1038/s41467-024-53241-1	Documents that do not assess or propose potential OECMs
61	Fanelli, E; Dell'Anno, A; Nepote et al.	Positive effects of two decades of passive ecological restoration in a historically polluted marine site	2023	*Frontiers in Marine Science*	10	-	1199654	10.3389/fmars.2023.1199654	Documents that do not assess or propose potential OECMs
62	Crowe, O; Beresford, AE; Buchanan, GM et al.	A global assessment of forest integrity within Key Biodiversity Areas	2023	*Biological Conservation*	286	-	110293	10.1016/j.biocon.2023.110293	Documents that do not assess or propose potential OECMs
63	Fidelino, JS; Constantino, RRP; Duya, MRM	Cross-taxon congruence of terrestrial vertebrates across Philippine Key Biodiversity Areas	2025	*Journal for Nature Conservation*	86	-	126907	10.1016/j.jnc.2025.126907	Documents that do not assess or propose potential OECMs
64	Yang, L; Chen, T; Zhang, L et al.	Overlap between priority conservation areas and natural assets appeals to a shared responsibility for global primate conservation	2024	*Global Ecology and Conservation*	54	-	e03124	10.1016/j.gecco.2024.e03124	Documents that do not assess or propose potential OECMs
65	Saensouk, P; Saensouk, S; Boonma, T et al.	Ethnomedicinal Properties of Wild Edible Fruit Plants and Their Horticultural Potential Among Indigenous Isan Communities in Roi Et Province, Northeastern Thailand	2025	*Horticulturae*	11	-	885	10.3390/horticulturae11080885	Documents that do not assess or propose potential OECMs
66	Wang, YH; Zhang, CC; Qiu, L et al.	Gaps in mammal conservation in China: An analysis with a framework based on minimum area requirements	2023	*Global Change Biology*	29	-	5224–5239	10.1111/gcb.16843	Documents that do not assess or propose potential OECMs
67	Gerstner, BE; Zarnetske, PL	Evaluating the effectiveness of protected areas and community-managed lands in capturing multiple dimensions of frugivorous biodiversity in the Tropical Andes	2025	*Biological Conservation*	302	-	110904	10.1016/j.biocon.2024.110904	Documents that do not assess or propose potential OECMs

68	Scalbert, M; Fonteyn, D; Houngbégnon, F et al.	Short-term impacts of selective logging on forest elephants	2025	*Conservation Science and Practice*	7	2	e13300	10.1111/csp2.13300	Documents that do not assess or propose potential OECMs
69	Campbell, M; Samhouri, JF; White, JW	Modeling consequences of spatial closures for offshore energy: Loss of fishing grounds and fishery-independent data	2025	*Ecosphere*	16	7	e70336	10.1002/ecs2.70336	Documents that do not assess or propose potential OECMs
70	Smit, KP; Sink, KJ; Shannon, LJ et al.	Groundtruthing cumulative impact assessments with biodiversity data: Testing indicators and methods for marine ecosystem condition assessments in South Africa	2024	*Aquatic Conservation: Marine and Freshwater Ecosystems*	34	2	e4096	10.1002/aqc.4096	Documents that do not assess or propose potential OECMs
71	van Rees, CB; Geist, J; Arthington, AH	Grasping at water: a gap-oriented approach to bridging shortfalls in freshwater biodiversity conservation	2025	*Biological Reviews*	100	-	1970–1993	10.1111/brv.70030; 10.1111/brv.70030	Documents that do not assess or propose potential OECMs
72	Duarte, GT; Schuster, R; Edwards, M et al.	Flood prevention benefits provided by Canadian natural ecosystems	2024	*Ecosystem Services*	70	-	101670	10.1016/j.ecoser.2024.101670	Documents that do not assess or propose potential OECMs
73	Zhang, MX; Liu, XY; He, WM et al.	From isolation to connectivity: A graph theory approach for optimising karst protected areas using an umbrella species	2025	*Biological Conservation*	309	-	111295	10.1016/j.biocon.2025.111295	Documents that do not assess or propose potential OECMs
74	Olafsdóttir, GA; Henke, T; Chambers, CP; Olafsdóttir, SH	Gaps in legislation and communication identified as stakeholders reflect on 30 x 30 policy in Icelandic waters	2024	*Marine Policy*	170		106422	10.1016/j.marpol.2024.106422	Documents that do not assess or propose potential OECMs
75	J.F., Brodie, Jedediah F.; M.C.M., Deith, Mairin C.M et al.	The contribution of other effective area-based conservation measures (OECMs) to protecting global biodiversity	2025	*Nature Communications*	16	-	7886	10.1038/s41467-025-63205-8	Documents that do not assess or propose potential OECMs

76	H., Ren, Hai; R.T., Corlett, Richard T et al.	How can China protect 30% of its land?	2025	*Trends in Ecology & Evolution*	40	9	824-826	10.1016/j.tree.2025.06.014	Documents that do not provide sufficient details on potential OECMs
77	M.A., Cruz, Margaux Angelica	A Geospatial Approach for the Assessment and Management Prioritization of Philippine Terrestrial Key Biodiversity Areas: Towards Meeting Global Sustainability Targets	2025	*ISPRS Annals of the Photogrammetry, Remote Sensing and Spatial Information Sciences*	X-G-2025	-	205–212	10.5194/isprs-annals-X-G-2025-205-2025	Documents that do not assess or propose potential OECMs
78	B.L., Jones, Benjamin L.; L., Coals, Lucy; L.C., Cullen-Unsworth, Leanne C. et al.	Mapping global threats to seagrass meadows reveals opportunities for conservation	2025	*Environmental Research Ecology*	4	-	25005	10.1088/2752-664X/adcacb	Documents that do not assess or propose potential OECMs
79	P., Sethi, Pia; N.P., Broome, Neema Pathak; G., Shahabuddin, Ghazala	OECMs Lost Opportunity for Inclusive Conservation?	2025	*Economic & Political Weekly*	LX	19	13-17		Documents that do not provide sufficient details on potential OECMs
80	X., Wang, Xiaoqian; Y., Deng, Yi	Key issues and advancement strategies for China's OECMs in alignment with the Kunming-Montreal Global Biodiversity Framework	2025	*Biodiversity Science*	33	3	24569	10.17520/biods.2024569	Documents published in languages other than the reviewers' language competency
81	W., Zhao, Weiyang; W., Wang, Wei; B., Ma, Bingran	Advances and prospects in research on other effective area-based conservation measures (OECMs)	2025	*Biodiversity Science*	33	3	24525	10.17520/biods.2024525	Documents published in languages other than the reviewers' language competency
82	X., Tang, Xiaoping; H., Tian, He; Y., Jiang, Yafang; B., Zhang, Bolin	Systematic Optimization of In-situ Biodiversity Conservation Patterns; 系统优化生物多样性就地保护格局	2024	*Research of Environmental Sciences*	37	10	2093-2099	10.13198/j.issn.1001-6929.2024.09.07	Documents published in languages other than the reviewers' language competency

83	J.A., Fitzsimons, James A.; S., Stolton, Sue; N., Dudley, Nigel; B.A., Mitchell, Brent A.	CLARIFYING 'LONG-TERM' FOR PROTECTED AREAS AND OTHER EFFECTIVE AREA-BASED CONSERVATION MEASURES (OECMS): WHY ONLY 25 YEARS OF 'INTENT' DOES NOT QUALIFY	2024	*Parks*	30.1	-	89-93	10.2305/GLFT9809	Documents that do not assess or propose potential OECMs
84	M., Fujino, Masaya; K., Oyama, Kohei	Examining the Challenges of Registering University Campuses as OECMs: A Case Study of Fukushima University Kanayagawa Campus.; 大学キャンパスの OECM 登録に向けた課題の検討—福島大学金谷川キャンパスを事例として—＊, ＊＊	2024	*J Jpn For Soc.*	106	3	68–74	10.4005/jjfs.106.68	Documents published in languages other than the reviewers' language competency
85	A.R., Paterson, Alexander Ross	Other Effective Area-Based Conservation Measures, Biodiversity Stewardship and Statutory Intervention – A South African Perspective	2023	*PER / PELJ*	26	-	1-31	10.17159/1727-3781/2023/v26i0a15441	Documents that do not assess or propose potential OECMs
86	M., Grbec, Mitja; T., Scovazzi, Tullio; I., Tani, Ilaria	Legal Aspects of Marine Protected Areas in the Mediterranean Sea: An Adriatic and Ionian Perspective	2023	*Routledge*	-	-	1-246	10.4324/9781003367963	Documents that do not assess or propose potential OECMs
87	I., Tani, Ilaria	Transboundary Area-based Conservation beyond the Territorial Sea within the Mediterranean Sea and the Adriatic and Ionian Seas	2023	*Routledge*	-	-	133-165	10.4324/9781003367963-6	Documents that do not assess or propose potential OECMs
88	Howard, Bui;	Other effective area-based conservation measures (OECMs) for the conservation and wise use of wetlands		*Convention on Wetlands*	-	-	1-25	10.69556/strp.bn14.25.eng	Documents that do not assess or propose potential OECMs

89	Charles, Anthony;	Human Dimensions of Fishery-Focused OECMs (Other Effective area-based Conservation Measures)	2024	-	-	-	-		Documents that do not assess or propose potential OECMs
90	Shim, Yun-Jin; Sung, Jung-Won; Lee, Kyeong-Cheol et al.	Site-level assessment of other effective area-based conservation measures-Focusing on the Korea national arboretum	2023	*J. Korean Env. Res. Tech.*	26	2	1-11	-	Documents published in languages other than the reviewers' language competency
91	Johnson, David E; Froján, Christopher Barrio; Diz, Daniela et al.	Other Effective Conservation Measures in the Marine Environment: The Policy-Makers' Silver Bullet for Meeting Global Conservation Ambitions?	2025	*Journal of Coastal Research*	-	113	16-20	10.2112/JCR-SI113-004.1	Documents that do not assess or propose potential OECMs
92	Jung, Na-Young; Oh, Choong-Hyeon;	Site-Level Assessment of Other Effective Area-based Conservation Measures for Temple Forests-Focusing on the Yeongwol Beopheungsa temple forest	2025	*J. Korean Env. Res. Tech.*	28	3	25-39	-	Documents published in languages other than the reviewers' language competency
93	Agardy, T; Himes-Cornell, A; Bowser, L; Hoelting, K;	Documenting biodiversity outcomes in marine fisheries management: A supplemental guide to the FAO handbook on identifying, evaluating, and reporting fisheries other effective area-based conservation measures	2025	*FAO Fisheries and Aquaculture Technical Paper*	-	709	-	10.4060/cd6085en	Documents that do not assess or propose potential OECMs
94	Lee, Da-Young;	Exploring Other Effective Conservation Measures (OECMs) for natural heritage sites-Focusing on the Dansanmok and Dansanje in Establishing the national biodiversity strategy and action plan	2023	*Journal of the Korean Institute of Traditional Landscape Architecture*	41	3	27-46	-	Documents published in languages other than the reviewers' language competency
95	Flitcroft, Rebecca; Abell, Robin; Moberg, Tara et al.	The role of OECMs for inland water biodiversity outcomes	2024	*IUCN WCPA Technical Note*	-	17	5		Documents that do not assess or propose potential OECMs

96	Oh, Ju-Hyeong; Kim, Su-Jin; Kim, Tae-Su et al.	Comparison between village characteristics and habitat quality to application OECM in Nakdong-Jeongmaek	2023	*J. Korean Env. Res. Tech.*	26	6	51-65	-	Documents published in languages other than the reviewers' language competency
97	Lee, Seonmi; Moon, Yoonjung; Cha, Jaegyu;	Enhancing connectivity and expanding the area of the Baekdudaegan Protected Area to achieve Kunming-Montreal GBF goals	2024	*J. Environ. Impact Assess*	33	6	335-351	-	Documents published in languages other than the reviewers' language competency
98	Heo, Hag-Young; Park, Sun-Joo;	A Study on the Identifying OECMs in Korea for Achieving the Kunming-Montreal Global Biodiversity Framework-Focusing on the Concept and Experts' Perception	2023	*Korean J. Environ. Ecol.*	37	4	302-314	-	Documents published in languages other than the reviewers' language competency
99	Kim, Kyou-Sub;	A Study on the Ecological and Cultural Conservation Strategies for Traditional Landscapes	2025	*Journal of the Korean Institute of Traditional Landscape Architecture*	43	1	29-38	-	Documents published in languages other than the reviewers' language competency
100	Shim, Yun-Jin; Sung, Jung-Won; Lee, Kyeong-Cheol et al.	A study on the site-level assessment criteria of OECM in Korea for achieving kunming-montreal global biodiversity framework-Focusing on the national Gariwangsan natural recreation forest	2024	*J. Korean Env. Res. Tech.*	27	2	17-28		Documents published in languages other than the reviewers' language competency
101	Shim, Yun-Jin; Sung, Jung-Won; Lee, Kyeong-Cheol et al.	Study on the Potential Identification of OECM in Ecological Restoration Sites-Focusing on National Geombongsan Natural Recreational Forest	2025	*J. Korean Env. Res. Tech.*	28	2	25-36	-	Documents published in languages other than the reviewers' language competency
102	최지원; 오충현;	서울시 보호지역 확대를 위한 지역기반 보전수단 (OECM) 도입방안	2023	-	-	-	-	-	Documents published in languages other than the reviewers' language competency

103	허학영; 박선주;	유네스코 생물권보전지역 (Biosphere Reserve) 의 기타 효과적인 보전수단 (OECM) 등재 가능성 검토 연구	2023	-	-	-	-	-	Documents published in languages other than the reviewers' language competency
104	Jung, Na-Young; Oh, Choong-Hyeon;	A Study on the Designation of Temple Forests as OECMs to Support GBF 2030	2025	*Korean J. Environ. Ecol*	39	1	102-111		Documents published in languages other than the reviewers' language competency
105	Solihin, Akhmad; Kushardanto, Hari; Purnama, Ray Chandra;	Urgensi Penyusunan Kerangka Hukum Pelaksanaan Other Effective Area-Based Conservation Measures (OECM) di Perairan Indonesia	2024	*Policy Brief*	6	4	-		Documents published in languages other than the reviewers' language competency

Table S6. *Database containing the full set of data extracted by the Scoping Review.*

See Supplementary file 2

Table S7. *List of sector and region-specific guidance documents relevant to OECMs.*

**Organisation/ Agency / Authority**	**Author(s)**	**Year**	**Title**	**Link**
IUCN	IUCN-WCPA Task Force on OECMs	2019	Recognising and reporting other effective area-based conservation measures	https://portals.iucn.org/library/node/48773
ICES	ICES	2021	ICES/IUCN-CEM FEG Workshop on Testing OECM Practices and Strategies (WKTOPS)	https://ices-library.figshare.com/ndownloader/files/37052161
Southeast Asia Rainforest Research Partnership - SEARRP	Southeast Asia Rainforest Research Partnership - SEARRP	2021	Recognising and Reporting OECMs in Malaysia	https://projects.searrp.org/static/oecm-malaysia/; https://projects.searrp.org/static/wp-content/uploads/2020/06/OECM-Site-based-Assessment-Phase_ENG.pdf
EBCD, IUCN/ CEM/ FEG	Garcia S, Rice J, Charles A, Diz D	2021	Other Effective Area-based Conservation Measures In Marine Capture Fisheries Systematic approach to Identification, use and performance assessment (Version 2)	https://ebcd.org/wp-content/uploads/2022/05/2021-Garcia-et-al-Systematic-approach-WKTOPS-ver-2.1-corrected-1.pdf
UNEP-WCMC, IEEP & Trinomics	UNEP-WCMC, IEEP & Trinomics	2021	Assess the potential of other effective area-based conservation measures as a driver for landscape-level conservation and connectivity in the EU Report	https://biodiversity.europa.eu/europes-biodiversity/protected-areas-archived/final_report_oecms_in_eu_submitted_2021031.pdf
Government of Canada - Fisheries and Ocean Canada (DFO)	Fisheries and Oceans Canada	2022	Government of Canada guidance for recognizing other effective area-based conservation measures in the marine environment.	https://www.dfo-mpo.gc.ca/oceans/oecm-amcepz/index-eng.html
Indian Ministry of Environment, Forest and Climate Change (MoEFCC), National Biodiversity Authority of India (NBA) and United Nations Development Programme (UNDP)	UNDP	2022	Criteria and Guidelines for Identifying Other Effective Area Based Conservation Measures (OECMs) in India	https://www.undp.org/sites/g/files/zskgke326/files/2022-06/OECM_criteria%20and%20guidelines_India_May%202022_.pdf

FAO	FAO	2022	A handbook for identifying, evaluating and reporting other effective area-based conservation measures in marine fisheries	https://openknowledge.fao.org/items/a6d2fcd6-76ff-4535-96f8-73d706380bc7
Ministry of Environment, Climate Change and Technology Male', Maldives	Ministry of Environment, Climate Change and Technology Male', Maldives	2022	Guideline for Recognising Areas as Other Effective Area-based Conservation Measures (OECMs) in Areas Leased for Tourism Operations	https://www.environment.gov.mv/v2/wp-content/files/publications/20220428-pub-guideline-oecm-en.pdf
IUCN	Comité français de l'UICN	2022	Recommendations for a future implementation of the OECM concept in France	https://uicn.fr/les-autres-mesures-de-conservation-par-zone/
European Commission	EC	2022	COMMISSION STAFF WORKING DOCUMENT - Criteria and guidance for protected areas designations	https://environment.ec.europa.eu/system/files/2022-01/SWD_guidance_protected_areas.pdf
Forum for the Conservation of the Patagonian Sea and Areas of Influence	Forum for the Conservation of the Patagonian Sea and Areas of Influence	2022	About Other Effective Area-Based Conservation Measures (OECMs)	https://marpatagonico.org/descargas/OMEC_Eng.pdf
HELCOM	HELCOM	2023	Regional common understanding of the CBD criteria for Other Effective Area-based Conservation Measures (OECMs)	https://helcom.fi/wp-content/uploads/2023/06/Regional-common-understanding-of-the-OECM-criteria-and-potential-OECM-identification-tree.pdf
UNEP-WCMC, Cook Islands National Environmental Service, Secretariat of the Pacific Regional Environmental Pregramme, IUCN Oceania	B. Lucas, J. Nicholson, A. Lewis, S. Whatarau, H. Weeks, M. Herman, E. Munro, V. Jungblut, J. Upton, J. Lessman, Hl Bingham, R. Chand	2023	Other Effective Area-Based Conservation Measures (OECMs) in the CookIslands	https://environment.gov.ck/wp-content/uploads/2023/12/OECMs-in-the-Cook-Islands_FINAL-1.pdf
Quebec Votre Gouvernement	Quebec Votre Gouvernement	2024	Recognizing Other Effective Conservation Measures (OECM) Within Continental Québec	https://www.environnement.gouv.qc.ca/biodiversite/aires_protegees/amce/lignes-directrices-AMCE-en.pdf

Australian Government - Department of Climate Change, Energy, the Environment	Department of Climate Change, Energy, the Environment and Water	2024	National Other Effective area-based Conservation Measures (OECMs) Framework	https://www.dcceew.gov.au/environment/land/achieving-30-by-30/conserved-areas/national-oecms-framework
IUCN	Jonas, H. D., Wood, P. & Woodley, S.,	2024	Guidance on other effective area-based conservation measures (OECMs)	https://portals.iucn.org/library/node/51773
IUCN	B. Herrera Fernández, J. Courrau Arias	2024	Other Effective Area-based Conservation Measures (OECMs): concept guide and guidelines for their identification and monitoring in Central America	https://thecpag.org/sites/default/files/thematic-resources/guidelines/other-effective-area-based-conservation-measures-oecms.pdf
North Sea Advisory Council - NSAC	NSAC	2024	NSAC Advice Ref. 01-2425 - NSAC Advice on Fisheries-OECMs	https://www.nsrac.org/wp-content/uploads/2024/11/01-2425-NSAC-Advice-on-Fisheries-OECMs.pdf
The Nature Conservacy & IUCN	The Nature Conservacy & IUCN	2024	Technical Guidance to Support the OECM Dialogue in Kenya	https://ke.chm-cbd.net/sites/ke/files/2024-12/IUCN-TNC%20OECM%20Technical%20Guidance_Kenya%20Nov%202024.pdf
Wales Environment Link	Wales Environment Link	2024	OECMs and their role in 30 by 30	https://waleslink.org/wp-content/uploads/2024/03/240221-WEL-Paper_-OECMs-role-in-30-x-30_-FINAL.pdf
WWF Nedbank Green Trust, BirdLife South Africa & Conservation Outcomes	Paterson A.	2024	Other Effective Area-Based Conservation Measures Legal Review – South Africa	https://law.uct.ac.za/sites/default/files/media/documents/law_uct_ac_za/1863/sa-oecm-legal-review-first-draft-draft-1-december-2024-with-toc-cover.pdf

Deutsche Zusammenarbeit, Federal Ministry for Economic Affairs and Climate Action, Interantional Climate Initiative, GIZ and IUCN	Deutsche Gesellschaft für Internationale Zusammenarbeit (GIZ) GmbH & IUCN	2024	Other Effective Area-Based Conservation Measures in Viet Nam:Heading Towards Recognition and Effective In-Situ ConservationBeyond Protected Areas	https://snrd-asia.org/wp-content/uploads/2025/02/OECMs-Summary-Final-En-31122024.pdf
IUCN	Stolpe, G., Howland, E. & Upton, J	2024	OECMS in Europe: The way forward	https://iucn.org/sites/default/files/2024-05/oecms-in-europe-final.pdf
IUCN	Sharma, M. and Pasha, M.K.S.	2024	Guidance to Advance Other Effective Area-Based Conservation Measures (OECMs) in Asia	https://iucn.org/sites/default/files/2024-03/guidance-to-advance-oecms-in-asia_feb2024-compressed.pdf
NTNU	Dunshea, G., Olaussen, K. & Eckbo, N.H.	2024	Potential marine Other Effective Area-Based Conservation Measures (OECMs) in Norway: Current compliance status in relation to CBD and IUCN guiding principles, definitions and criteria	https://www.ntnu.no/documents/10476/1350233483/2024-3+Rapport+OECM.pdf/4a75741d-b33d-6365-65f2-acc0368e8823?t=1716379306187
FAO	Agardy, T.; Himes-Cornell, A.; Bowser, L.; Hoelting, K.;	2025	Documenting biodiversity outcomes in marine fisheries management A supplemental guide to the FAO handbook on identifying, evaluating, and reporting fisheries other effective area-based conservation measures	https://openknowledge.fao.org/items/9fa8460b-d78f-4306-885d-b9aa07b224e7
Ministry of the Environment - Government of Japan	Ministry of the Environment - Government of Japan	2025	Japan's OECM and related policy - Nationally Certified Sustainably Managed Natural Sites	https://www.env.go.jp/nature/biodiversity/OECM.html
NatureScot	NatureScot	2025	Nature30 - The development of OECMs (Other Effective area-based Conservation Measures) in Scotland	https://www.nature.scot/professional-advice/protected-areas-and-species/30-30-and-nature-networks/nature30-development-oecms-other-effective-area-based-conservation-measures

Ramsar - Convention on Wetlands	Ramsar - Convention on Wetlands	2025	Other effective area-based conservation measures (OECMs) for the conservation and wise use of wetlands	https://www.ramsar.org/sites/default/files/2025-06/STRP_BN14_OECM_EN_v2.pdf
OSPAR	OSPAR	2025	OSPAR guidance on identifying and recognising Other Effective Area-Based Conservation Measures (OECMs) in the North-East Atlantic	https://www.ospar.org/documents?v=63650
WWF EU	WWF EU	2025	Beyond Marine Protected Areas (MPAs): Integrating Other Effective Area-Based Conservation Measures (OECMs) to strengthen EU Conservation Networks	https://wwfeu.awsassets.panda.org/downloads/policy-brief-on-oecm.pdf
Wales Biodiversity Partnership	Wales Biodiversity Partnership	2025	Initial recommendations of the 30 by 30 Other Effective Area-based Conservation Measures and Nature Recovery Exemplar Areas Expert Group for Wales	https://wagtail.biodiversitywales.org.uk/en/our-work/30-by-30-in-wales/other-effective-area-based-conservation-measures-oecms-and-nature-recovery-exemplar-areas-nreas/
UNEP RAC/ SPA	Wells S.	*In preparation*	Guidance Document on OECMs in the Mediterranean	https://spa-rac.org/en/article/258/agem-new-membership-and-a-guidance-document-on-oecms-in-the-mediterranean-under-preparation
International Indigenous Forum on Biodiversity - IIFB	International Indigenous Forum on Biodiversity - IIFB	*In preparation*	The Marine OECM Guide - A practical, inclusive tool for recognizing effective ocean conservation	https://iifb-indigenous.org/initiatives/marine-oecm-guide/
Oregon State University	Grorud-Colvert K.	*In preparation*	A Guide for Recognizing and Accelerating Effective Ocean Conservation	-

References

CBD. 2018. Protected areas and other effective area-based conservation measures. CBD/COP/DEC/14/8. Convention on Biological Diversity. https://www.cbd.int/doc/decisions/cop-14/cop-14-dec-08-en.pdf

Petza, D., E. Amorim, E.B. Lamine, F. Colloca, E.D. Crisóstomo, E. Fabbrizzi, S. Fraschetti, I. Galparsoro, et al. 2023. Assessing the potential of Other Effective area-based Conservation Measures (OECMs) for contributing to conservation targets: A global scoping review protocol. *Open Research Europe* 3:118. https://doi.org/10.12688/openreseurope.16116.3

Tricco A., E. Lillie, W. Zarin, K. O'Brien, H. Colquhoun, D. Levac, D. Moher, M.J. Peters, et al. 2018. PRISMA Extension for Scoping Reviews (PRISMA-ScR): Checklist and Explanation. Annals of Internal Medicine 169 (7): 467-467. https://doi.org/10.7326/M18-0850
